# Accelerating precision anti-cancer therapy by time-lapse and label-free 3D tumor slice culture platform

**DOI:** 10.7150/thno.59533

**Published:** 2021-09-13

**Authors:** Fuqiang Xing, Yu-Cheng Liu, Shigao Huang, Xueying Lyu, Sek Man Su, Un In Chan, Pei-Chun Wu, Yinghan Yan, Nana Ai, Jianjie Li, Ming Zhao, Barani Kumar Rajendran, Jianlin Liu, Fangyuan Shao, Heng Sun, Tak Kan Choi, Wenli Zhu, Guanghui Luo, Shuiming Liu, De Li Xu, Kin Long Chan, Qi Zhao, Kai Miao, Kathy Qian Luo, Wei Ge, Xiaoling Xu, Guanyu Wang, Tzu-Ming Liu, Chu-Xia Deng

**Affiliations:** 1 Cancer Center, Faculty of Health Sciences, University of Macau, Macau SAR, China.; 2 Centre for Precision Medicine Research and Training, Faculty of health Sciences, University of Macau, Macau SAR, China.; 3 MOE Frontier Science Centre for Precision Oncology, University of Macau, Macau SAR, China.; 4 Centre of Reproduction, Development and Aging, Faculty of Health Sciences, University of Macau, Macau SAR, China.; 5 Kiang Wu Hospital, Macau SAR, China.; 6 Department of Biology, School of Life Sciences & Guangdong Provincial Key Laboratory of Computational Science and Material Design, Southern University of Science and Technology, Shenzhen, China.

**Keywords:** 3D tumor slice culture, apoptosis, FRET technique, label-free, personalized medicine

## Abstract

The feasibility of personalized medicine for cancer treatment is largely hampered by costly, labor-intensive and time-consuming models for drug discovery. Herein, establishing new pre-clinical models to tackle these issues for personalized medicine is urgently demanded.

**Methods:** We established a three-dimensional tumor slice culture (3D-TSC) platform incorporating label-free techniques for time-course experiments to predict anti-cancer drug efficacy and validated the 3D-TSC model by multiphoton fluorescence microscopy, RNA sequence analysis, histochemical and histological analysis.

**Results**: Using time-lapse imaging of the apoptotic reporter sensor C3 (C3), we performed cell-based high-throughput drug screening and shortlisted high-efficacy drugs to screen murine and human 3D-TSCs, which validate effective candidates within 7 days of surgery. Histological and RNA sequence analyses demonstrated that 3D-TSCs accurately preserved immune components of the original tumor, which enables the successful achievement of immune checkpoint blockade assays with antibodies against PD-1 and/or PD-L1. Label-free multiphoton fluorescence imaging revealed that 3D-TSCs exhibit lipofuscin autofluorescence features in the time-course monitoring of drug response and efficacy.

**Conclusion**: This technology accelerates precision anti-cancer therapy by providing a cheap, fast, and easy platform for anti-cancer drug discovery.

## Introduction

Recent advances in novel anti-cancer drug discovery have afforded new insights into personalized precision oncology for individual patients instead of standard clinical therapeutic options 1, 2. Personalized cancer treatment requires reliable prediction of molecular response to chemotherapy in individual patients, which can prevent unwanted side effects sometimes associated with the therapy. Though next-generation sequencing (NGS) has been widely used in the past decade, it remains unable to identify therapeutic targets for the majority of patients with advanced disease 3. Thus, high-throughput drug screening followed by validation of top candidates is imperative for discovering effective pharmacologic treatments. Previous studies reported various models for anti-cancer drug discovery including cell-based assays 4, 5, zebrafish xenografts 6-8, patient derived xenografts (PDX) in mouse 9-11, and patient-derived organoids (PDOs) model 12-15. Given each method can be used for some purposes, it still has its own weakness and cannot replace each other in anti-cancer drug screening and toxicity studies 16, 17. For example, animal models for preclinical pharmaceutical research are time-consuming (months may be needed for generation of PDX), high cost, and labor-intensive for drug discovery. The PDOs faithfully mimic many key features in the primary cancers in mutation spectrum, gene expression and histopathology 18-22, but attempts to create organoids from small quantity of cancer biopsies sometimes might suffer from low number of cancer cells and low overall success rates due to the inability of cells to quickly adapt environmental change from tissue to the culture conditions 19, 23. Moreover, due to the lack of immune components, the PDOs system has limitations to be used for studying interactions between tumor cells and immune cells 24, 25. Therefore, establishing new pre-clinical models to tackle these issues for personalized medicine is urgently demanded.

Recently, tumor-fragment (or *ex vivo* tumor slice) culture-based assay has been used to test the sensitivity of anti-cancer drugs, which aims to quickly identify drugs for personalized therapy 26-32. The prototype of tumor slice culture was first reported in 2013 by Holliday et al. for studying the response of breast slices to tamoxifen or doxorubicin 33. However, this method is still at its early stages of development, and its feasibility remains unclear due to some limitations. First, it is more difficult to quantitatively evaluate the drug sensitivity and efficiency in tumor fragments than PDOs due to the lack of knowledge about the impact of slicing on tumor tissue heterogeneity, integrity and immune microenvironment 31. Second, due to the limited number of slice preparation, the tumor slice culture could only screen fewer drugs each time compared with 2-D cell culture and PDO model 34. Third, conventional fixation and dye labeling evaluation may neglect the cytomic dynamics and metabolic shift in the microenvironment of drug treatment. This pharmacodynamic information of organoids may provide valuable indication to the therapeutic efficacy.

To overcome these shortcomings, we have integrated histochemical staining, transgenic fluorescent reporter, and label-free metabolic imaging approaches to time-course visualize the pharmacodynamics and quantitatively evaluate the efficacy of anti-cancer drugs in a 3-dimensional tumor slice culture (3D-TSC) model. 3D-TSC model mimics the tumor microenvironment and could be adapted to assess individualized drug responses and immune checkpoint blockade therapy within 4-7 days for surgically removed cancers.

## Results

### 3D-TSCs maintain the cell repertoire and immune components of their original tumor

**Figure 1:**
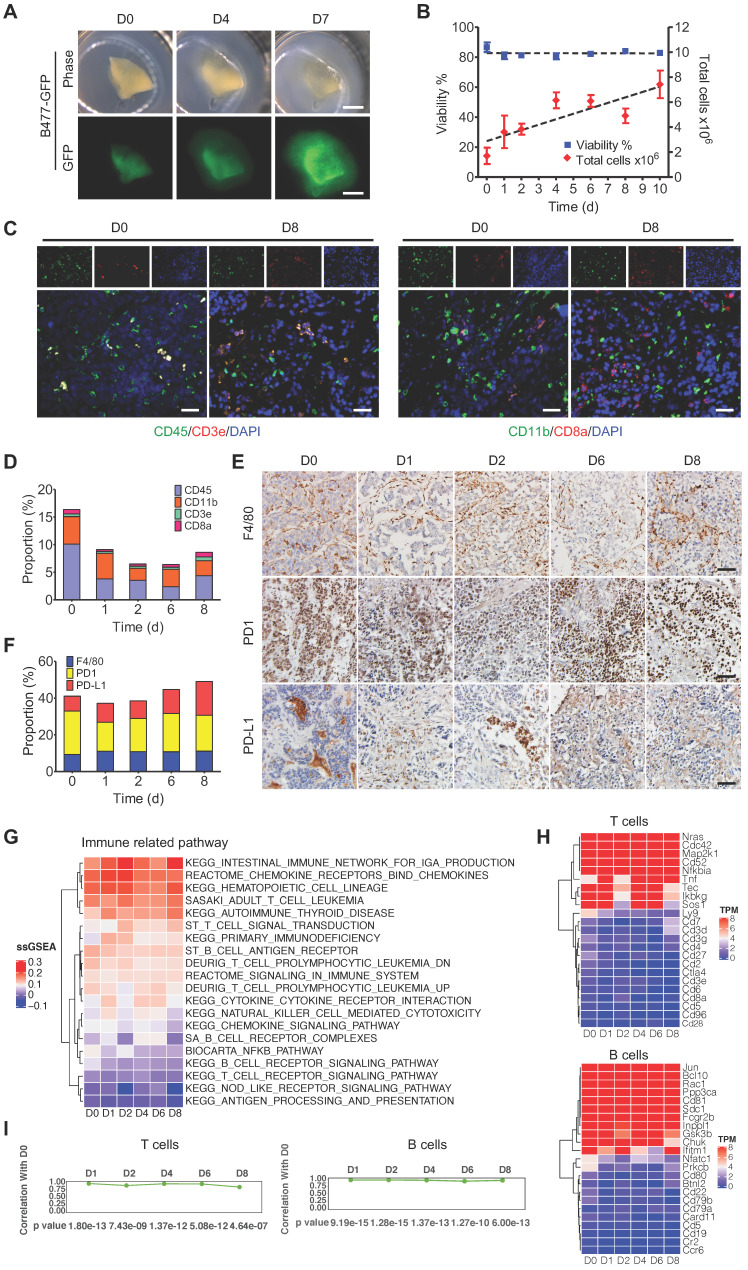
** 3D-TSCs maintain cell repertoire and immune components of its original tumor. A** Observation of tumor growth in 3D-TSCs derived from primary tumor formed by B477-GFP cells injected into the mammary fat pad of nude mice. Scale bar: 1 mm. **B** Time-lapse of cell viability in 3D-TSCs derived from B477-GFP mouse tumor.** C, E** Histological analysis of immune biomarkers in 3D-TSCs derived from genetically engineered *Brca1^Co/Co^;MMTV-Cre* mouse in immunocompetent hosts. Blue is nuclear counterstain by Hematoxylin, and brown staining is positive protein by DAB. CD3e/CD8a: T lymphocytes; CD11b, macrophages and microglia marker; CD45, T, NK, dendritic, and lymphokine-activated killer (LAK) cells marker; CD11b, macrophages and microglia; F4/80, mouse macrophage marker; PD-1, an immunoreceptor expressed by T cells; PD-L1, ligand of PD-1 expressed by myeloid, lymphoid, normal epithelial cells or cancer cells; Scale bar: 100 µm. **D, F,** Quantitation of immune biomarkers in 3D-TSCs derived from genetically engineered *Brca1^Co/Co^;MMTV-Cre* mouse in immunocompetent hosts. 5 digital images of antibody-stained tissue slides were captured for calculating the mean in each sample. **G** RNA sequence analysis of immune marker gene expression for immune related pathway. Single sample GSEA (ssGSEA) sore represents the level of the gene set is up- or down-regulated within a sample. **H** RNA sequence analysis of immune marker gene expression for T cells and B cells. Transcripts per million (TPM) is used to estimate transcript or gene expression levels. **I** Comparison of the gene expression level by calculating the Pearson correlation coefficient for T cells and B cells from D1 to D8 with D0.

To establish a platform for rapid and time-lapse prediction of response to anti-cancer drugs, we exploited a 3D-TSC system, in which the fresh tumors were embedded with agarose and sectioned at a thickness of 300 µm with a vibrating microtome. The tumor slices were then placed onto an air-liquid interface system for culturing (Figure S1), and time-course images of the slices were captured from original tumor prior to culture (referred to day 0, D0) to D 7 in culture using a Leica M165FC fluorescent stereo microscope. Under our culture conditions, the tumor slices derived from mouse mammary tumor B477-GFP grew bigger, and the GFP signal became stronger, within 7 days (Figure 1A). In order to further determine whether 3D-TSCs could mimic tumor growth, the model was validated using various methods. First, we monitored cell viability of 3D-TSCs by counting alive cells and found that total cell numbers gradually increase, while cell viability remains at >80% within 10 days (Figure 1B). Next, histological analysis of 3D-TSCs and various biomarkers, including γH2AX, Ki67, cleaved caspase-3, and desmin was performed (Figure S2). The data revealed that the 3D-TSCs, in general, exhibited features similar to those of primary tumors, except for increased Ki67+ cells (Figure S2A,B), which is consistent with increased proliferating cells observed earlier (Figure 1B). Staining with antibodies against E-cadherin and vimentin, markers for epithelial and mesenchymal cells, respectively, and against CK7, a protein that maintains the tumor parenchyma, also detected no significant differences during the culture time-course for up to 8 days.

To better understand the status of gene expression, transcriptome analysis by RNA-seq was conducted on 3D-TSCs at different time-points. The data revealed no significant changes in gene expression in general during the course of culture from D0 to D8, although some variations were observed (Figure S3A,B). This variation might be primarily caused by the varying number of cells at different phases in the cell cycle at different time point as we did not synchronize cells prior to RNA-sequencing. Next, we sought to determine whether 3D-TSCs could also maintain the tumor immune microenvironment of the original tumor. Thus, immune components were analyzed in 3D-TSCs obtained from endogenous mammary tumors derived from genetically engineered mouse model carrying mammary specific disruption of Brca1 (*Brca1^Co/Co^;MMTV-Cre*) 35. Immune markers were visualized by histological analysis, including CD3e, CD8a, CD11b, CD45, F4/80, PD-1, and PD-L1 (Figure 1C-E and Figure S3C). All of these immune markers were positive in 3D-TSCs during the cultured period of 8 days. Also, no significant differences were observed when compared with D0 (fresh tumor slice), suggesting that immune components are well preserved under slice culture conditions. RNA-sequencing showed a steady expression of immune genes in 3D-TSCs within the 8-day study period (Figure 1G,H and Figure S3D-G). Quantification of pathway genes expression levels from original tumor prior to culture D0, immediate after culture D1 to D8 by calculating the Pearson correlation coefficient for macrophages, neutrophils, monocytes, NK cells, T cells and B cells from D1 to D8 with D0, which showed high correlation (Figure 1I, Figure S3H). Altogether, our results indicate that 3D-TSCs maintain the cell repertoire and immune components of their original tumor.

### Quantitative and time-lapse prediction of the response of 3D-TSCs to anti-cancer drugs *ex vivo*

**Figure 2:**
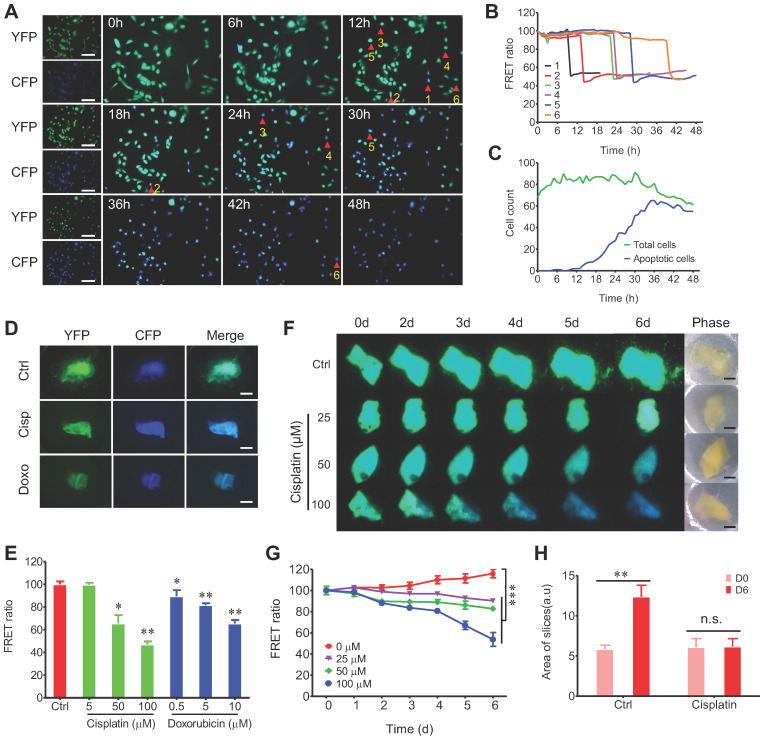
** FRET-based time-lapse predicting response of 3D-TSCs to anti-cancer drugs* ex vivo*. A** Time lapse observation of cell apoptosis in MDA-MB-231-C3 cells induced by 10 µM DOX within 48 h, CFP (ex: 430 nm/em: 480 nm) was merged with YFP (ex: 430 nm/em: 520 nm). Scale bar: 100 µm; **B** Quantification of FRET changes of single cell; **C** Analysis of DOX induced cell apoptosis in MDA-MB-231-C3 cells. Green and blue lines indicate total and apoptotic cells, respectively.** D** Cisplatin/doxorubicin induced cell apoptosis in 3D-TSC; MDA-MB-231-C3 tumor slices were treated with 100 µM cisplatin or 10 µM doxorubicin for six days. Scale bar: 1 mm. **E** Quantification of FRET ratio of 3D-TSCs. n = 3. **F** Dynamic courses of cell apoptosis in 3D-TSCs during various dose of cisplatin treatment. Scale bar: 1 mm. **G** Quantification of FRET ratio of 3D-TSCs in experiment of panel **(F)**. n = 4. **H** Quantification of area of 3D-TSCs after treatment with 100 µM cisplatin for six days. n = 4, error bars ± SEM, *p < 0.05; **p < 0.01; ***p < 0.001 (control versus indicated drug).

The establishment of 3D-TSC as a reliable model for drug sensitivity testing requires the quantitative measurement of cell death. Therefore, a caspase-3 reporter sensor C3 that express the CFP-Asp-Glu-Val-Asp (DEVD)-YFP fusion protein 36, 37 was introduced to cancer cell lines (Figure S4). The amino acid sequence DEVD is the cleavage site of caspase-3. Cells expressing sensor C3 can transfer electronic energy from CFP to YFP, resulting in green fluorescence emitted by the cells, so-called fluorescence resonance energy transfer (FRET) in live sensor cells. However, when the caspase-3 is activated, it cleaves DEVD between the fluorescent proteins, thereby eliminating the transfer of energy and resulting in emission of blue fluorescence from the cells. Therefore, the C3 sensor allows us to monitor the dynamics of caspase-3 during apoptosis in live cells (Figure 2A-C). Our time-course study revealed that C3 labeled cancer cells is more powerful in time-lapse evaluation of drug efficacy than conventional antibody-based caspase-3 activation assay or apoptosis staining approaches (such as TUNEL assay), which only monitor the endpoint and unable to detect early drug response of the culture.

Next, we tested whether time-lapse testing of the drug response in 3D-TSCs could also be achieved. Treating 3D-TSCs with cisplatin or doxorubicin led to a decrease of the FRET ratio in a dose-dependent manner (Figure 2D, E). Another time-course experiment had also revealed a decrease of the FRET ratio in a dose and time-dependent manner (Figure 2F-H). To verify apoptotic cells in 3D-TSCs, trypan blue and propidium iodide (PI) staining was performed on 3D-TSCs derived from MDA-MB-231-C3 tumor after drug treatment and detected increased trypan blue dye exclusion staining and fluorescence intensity of PI (Figure S5). These observations suggest that the drugs were responsible for the increased apoptosis seen in 3D-TSCs.

### FRET-based time-lapse and MTT endpoint assays to predict the efficacy of chemotherapy in 3D-TSCs

**Figure 3:**
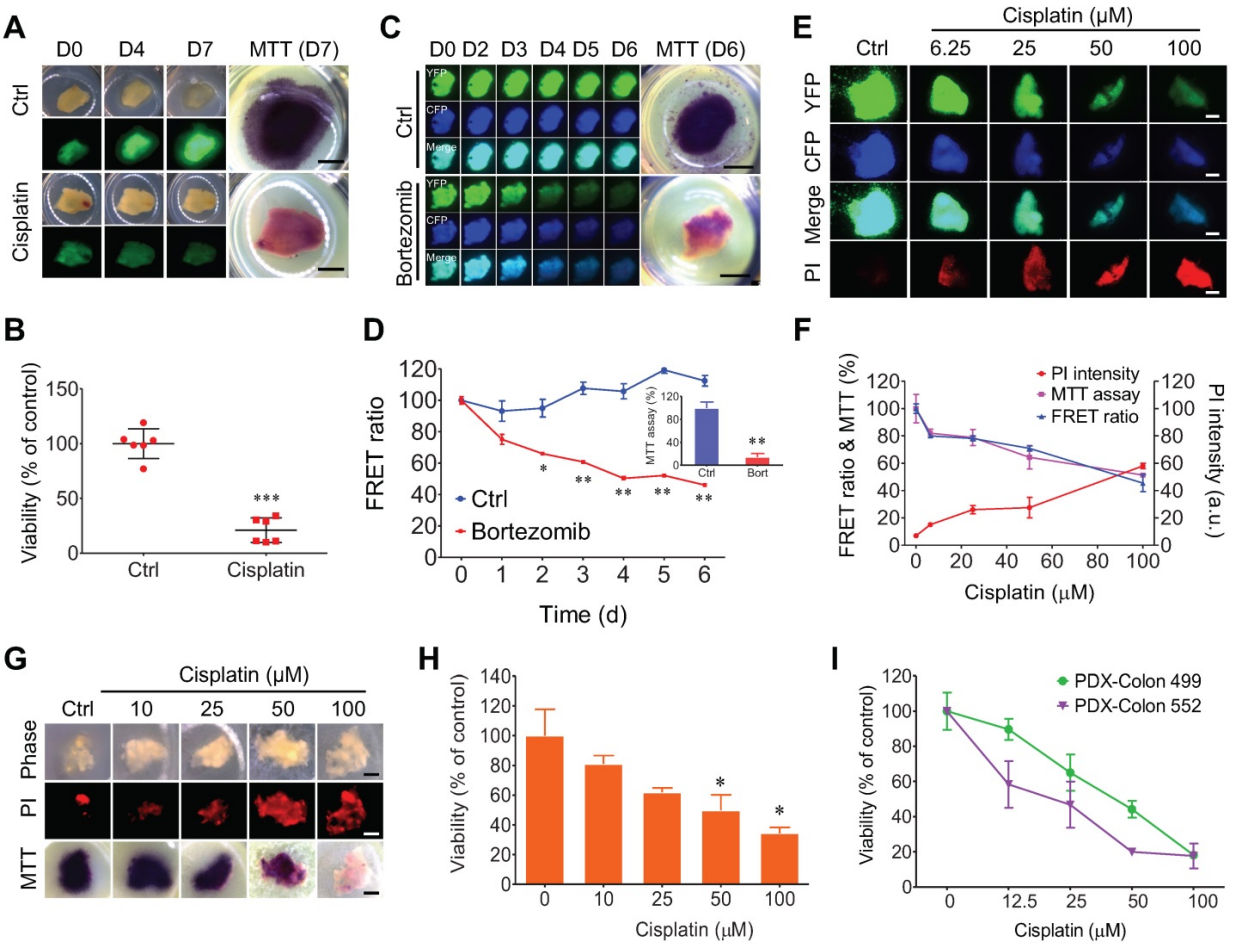
** Quantitative evaluating response of 3D-TSCs to anti-cancer drugs by MTT assay. A** Imaging of B477-GFP breast tumor slices treated or not treated with 100 µM cisplatin for 0-7 days by GFP fluorescence and MTT assay. Scale bar: 1mm. **B** Evaluation of drug efficacy in (**A**) at day 7 by MTT assay. n = 6. **C** Imaging of MDA-MB-231-C3 breast tumor slices treated with 1 µM bortezomib for six days by FRET fluorescence (d0-6) and MTT assay (d6).** D** Evaluation of drug efficacy in (**C**) by FRET ratio (d0-6) and MTT assay (d6). Scale bar: 2 mm. n = 3. **E, F** Evaluation of drug efficacy in MDA-MB-231-C3 breast tumor slices treated with various dose of cisplatin for six days by PI, FRET ratio and MTT assay. Scale bar: 1 mm. n = 4.** G,** Imaging of B477 tumor slices treated with cisplatin for six days by PI and MTT assay. Scale bar: 0.8mm. **H** Quantification of drug efficacy in (**G**) by MTT assay. n = 3. **I** Evaluation of drug efficacy in PDX-Colon tumor slices treated with cisplatin for five days by MTT assay. n = 3, error bars ± SEM, *p < 0.05; **p < 0.01; ***p < 0.001 (control versus CIS/DOX).

Because clinical cancer samples are devoid of fluorogenic markers, we then used our GFP-labeled cells, C3-labeled cells, and non-fluorogenic labeled cells in 3D-TSCs to test drug responses and to compare them to the following seven methods: MTT assay, PI staining, Hoechst 33342, LIVE/DEAD® viability/cytotoxicity kit, caspase-3/7 green detection reagent, NucRed live 647, and LysoTracker® deep red (Figure 3A-F and Figure S5-7). Firstly, the viability of fluorogenic samples was measured in B477-GFP breast tumor slices treated with cisplatin (Figure 3A, B). Increased GFP signaling was found within 7 days during 3D-TSC, and this increase was blocked upon cisplatin treatment, as reflected by size, GFP intensity, and MTT assay. We next treated MDA-MB-231-C3 tumor slices with 1 µM bortezomib and measured the drug response during a time-course study. The FRET ratio was found to have decreased in a graded manner during bortezomib treatment (Figure 3C, D). The MTT assay of cell viability was quantitatively measured in bortezomib treated MDA-MB-231-C3 at day 6 (Figure 3D).

3D-TSCs derived from MDA-MB-231-C3 tumor were also treated with increasing doses of cisplatin for 6 days, which resulted in a reduced FRET ratio and greater PI staining, which is excluded from live cells with intact membranes, but which penetrates dead or damaged cells and binds to DNA and RNA by intercalating between their bases (Figure 3E, F). The cells were then quantified using the MTT assay, which revealed a decline in the quantity of cells similar to that of the reduced FRET. Of note, Hoechst 33342 staining of nuclei also detected reduced fluorescence in living 3D-TSCs (Figure S6A-F); however, reduced/negative signaling is usually difficult to measure relative to increased/positive signaling. Thus, this dye was excluded in subsequent experiments. These data indicate that both PI imaging and MTT assaying could yield comparable results from treatment with different drugs.

Since all clinical samples were not labeled, PI imaging and MTT assaying were used to test the drug response in mouse non-fluorogenic B477 and PDX-colon 499/552 tumor slices. All slices could be used to quantitatively measure cell viability (Figure 3G-I). Other kits were also used to measure cell viability of non-fluorogenic tumor slices, but these kits showed no significant differences after drug treatment, suggesting that these kits are not appropriate for the evaluation of drug efficacy in 3D-TSCs (Figure S6, 7).

### FRET-based time-lapse drug screening *in vitro* and *ex vivo*

Because the quantity of tumor slices prepared from each tumor is limited, a 3D-TSC system cannot be used to test a large quantity of drugs. The C3-mediated cell model was first used to screen a drug library and to identify a small quantity of anti-cancer drugs for further evaluation. From this library, 166 candidate compounds were screened (Table S1), the majority of which are FDA approved for the clinical treatment of various cancers and conducted drug screening using MDA-MB-231-C3, HepG2-C3, Hct116-C3, PANC1-C3, and A549-C3 cell lines (Table S2). Because many of the drugs suppress the growth of tumor cells with the IC_50_ (50% cell death) between 5-20 μM depending on cell lines 38, 39, we first used the concentration of all drugs at 20 µM for a time-course study from 0-96h to have quick test in the 5 cell lines (Figure S8A, B and Table S2, 4-5). For drugs that kill cancer cells tested with high efficacy, we further test them using lower doses and also at shorter time points, the results indicated a graded reduction of cell numbers and increased apoptosis (Figure S8C and Table S3). For drugs that could not achieve IC_50,_ the drugs will not be used further. By using this way, we aimed to have a quick assessment of the efficacy of these drugs for the cancer cells tested and select top candidate drugs for further test in the 3D-TSC model. These data illustrate a feature of the C3-labeled cell lines that these cell lines can yield could serve as sensitive tools for high-throughput, time-lapse screening on much larger drug collections.

**Figure 4:**
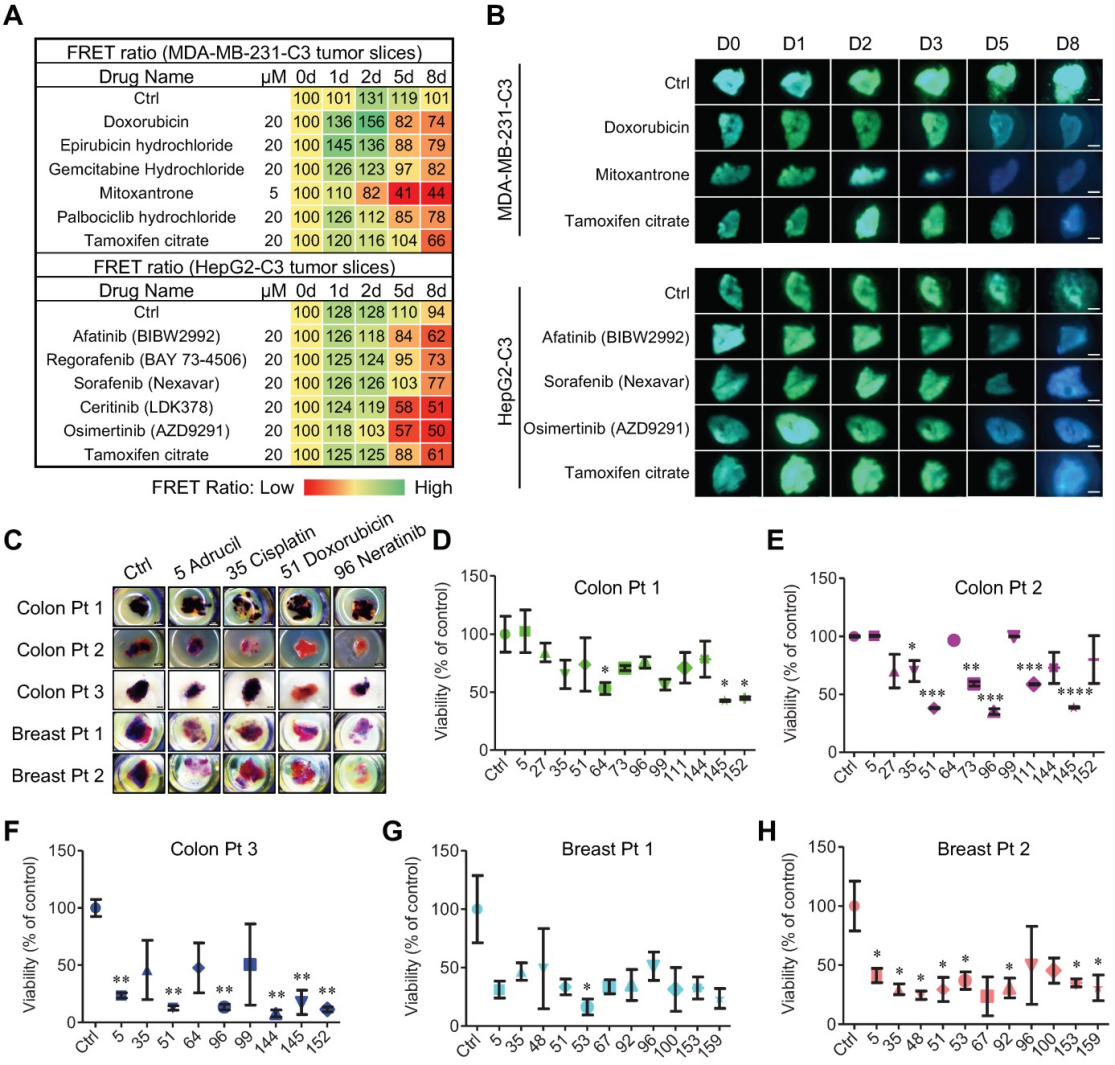
** FRET-based time-lapse and MTT endpoint predicting efficacy of chemotherapy in 3D-TSCs derived from mouse and human surgical tumors. A** Time lapse FRET evaluation of anti-tumor efficacy in 3D-TSCs. 3D-TSCs derived from MDA-MB-231-C3 and HepG2-C3 tumor were treated with indicated drugs. **B** Representative FRET images of 3D-TSCs treated with indicated drugs. Scale bar: 1 mm. **C** MTT staining of human colon and breast 3D-TSCs treated with drugs for 4 days. Scale bar: 1 mm. **D-F,** Drug response of human colon 3D-TSCs treated with drugs for 4 days by MTT assay. **D** (Colon Pt 1), **E** (Colon Pt 2), **F** (Colon Pt 3). The concentration of drugs used for human colon samples is 20 µM except for ceritinib (2.5 µM), daunorubicin hydrochloride (2.5 µM), osimertinib (5 µM). **G, H** Drug response of human breast 3D-TSCs treated with drugs for 4 days by MTT assay. **G** (Breast Pt 1), **H** (Breast Pt 2); The concentration of drugs used for human breast samples is 20 µM except for Mitoxantrone (5 µM) and Neratinib (10 µM). n = 3, error bars ± SEM, *p < 0.05; **p < 0.01; ***p < 0.001; ****p < 0.0001 (control versus indicated drug).

Based on high-throughput, time-lapse drug screening in C3 labeled cancer cell lines *in vitro*, we then evaluated the top candidate drugs in the 3D-TSC model. In order to screen compounds in 3D-TSCs, top drug candidates were selected to treat 3D-TSCs derived from 231-C3 and HepG2-C3 cells. In general, drugs that induce apoptosis *in vitro* were also found to be able to cause apoptosis in the 3D-TSCs model with varying efficiency (Figure 4A, B).

### Patient-derived tumor slice (PDTS) response to chemotherapy

3D-TSCs were then used as a model for evaluating drug efficacy of patient-derived tumor slice (PDTS). The PDTS derived from human colon cancer and breast cancer samples were treated with drugs that exhibit strong ability for apoptosis induction in C3-labeled colon and breast cancer cells (Figure 4C-H). We detected variations in individual cancers to the treatment during the course of 4-day treatment course. For colon cancer (Colon Pt 1), ftorafur, daunorubicin hydrochloride, and osimertinib significantly inhibited cell viability (Figure 4D), whereas for Colon Pt 2, cisplatin, doxorubicin, irinotecan, neratinib, regorafenib, and daunorubicin hydrochloride significantly inhibited cell viability (Figure 4E). The Colon Pt 3 sample exhibited strong sensitive to adrucil, doxorubicin, neratinib, ceritinib, daunorubicin hydrochloride, and osimertinib that led to ≤50% cell viability (Figure 4F). The drug efficacy was then measured by MTT assay in two human breast tumor samples, revealing that both were quite sensitive (Figure 4G, H). Though 9 out of 11 drugs tested led to ≤50% cell viability for the Breast Pt 1 sample, only the effect of epirubicin hydrochloride was statistically significant (Figure 4G). For the Breast Pt 2, 10 drugs (except neratinib) led to ≤50% cell viability, eight of which (adrucil, cisplatin, docetaxel, doxorubicin, epirubicin hydrochloride, mitoxantrone, palbociclib hydrochloride and tamoxifen citrate) were statistically significant (Figure 4H). These data highlight the need for personalized drug testing in precision medicine, for which our PDTS model may prove particularly useful.

### 3D-TSCs and PDTSs response to PD-1/PD-L1 checkpoint blockade

**Figure 5:**
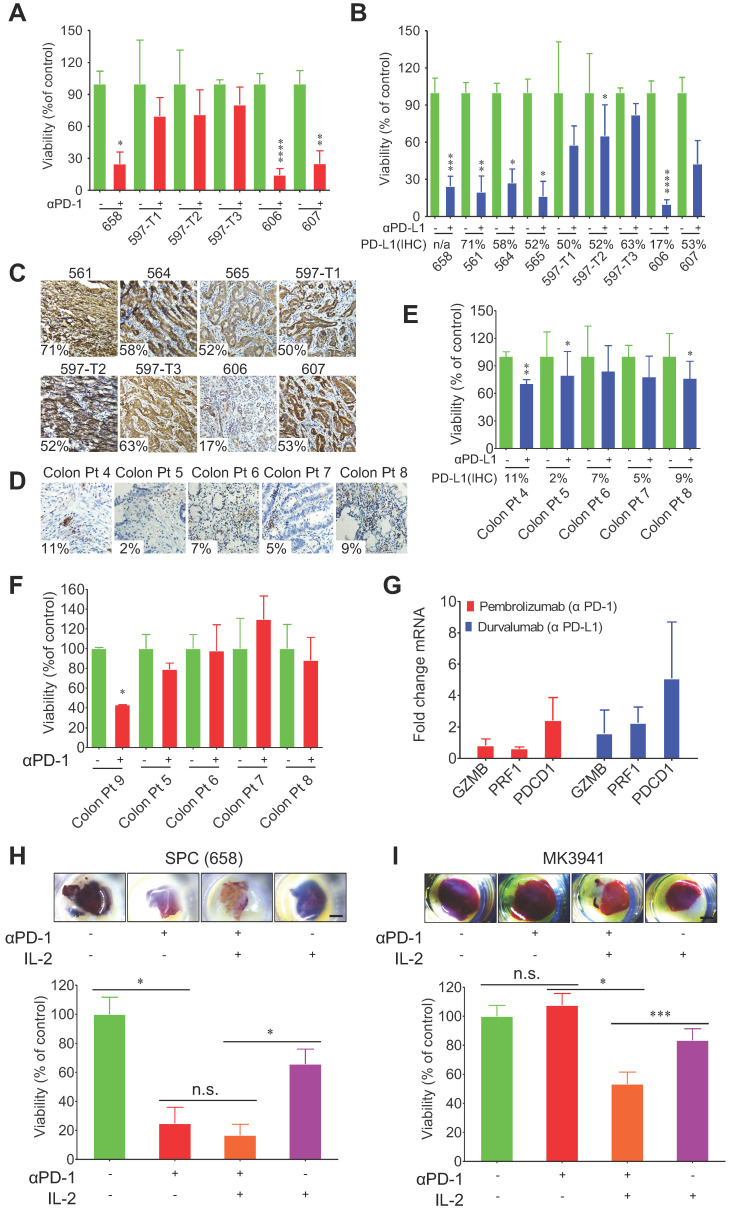
** 3D-TSCs response to anti-PD-1/PD-L1 checkpoint blockade. A** Anti-PD-1 induction of mouse 3D-TSCs cell death. MTT analysis of mouse *Smad4^co/co^;Pten^co/co^;Alb-Cre* (SPC) tumor slices (658-607) in immunocompetent hosts after 6 days control IgG or αPD-1 treatment, viability was normalized to control IgG. n = 3. **B** Anti-PD-L1 induction of mouse 3D-TSCs cell death. MTT analysis of mouse SPC tumor slices (658-607) in immunocompetent hosts after 6 days control IgG or αPD-L1 treatment, viability was normalized to control IgG. n = 3. Original tumor PD-L1 IHC % is depicted (n/a: original tumor not available for PD-L1 IHC). **C** Original tumor PD-L1 IHC staining of fresh tumor from (**B**). Original histology from one mouse samples (658) was not available for analysis. **D** Original tumor PD-L1 IHC staining of human surgical tumors. Original histology from human sample (Colon Pt 9) was not available for analysis.** E** Anti-PD-L1 induction of PDTS cell death. MTT analysis of human colon (Colon Pt 4- Colon Pt 7) tumor slices after 7 days control IgG1 or αPD-L1 (durvalumab) treatment, viability was normalized to control IgG. n = 3. Original tumor PD-L1 IHC % is depicted.** F** Anti-PD-1 induction of PDTS cell death. MTT analysis of human colon tumor slices (Colon Pt 7- Colon Pt 9) after 7 days control IgG4 or αPD-1 (pembrolizumab) treatment, viability was normalized to control IgG. n = 3.** G** qRT-PCR analysis of immune genes in human colon cancer samples (Colon Pt 4) after 7 days αPD-1/αPD-L1 treatment. n = 3.** H, I** Effect of IL-2 in immunotherapy of mouse 3D-TSCs derived from SPC tumor (658) and BRCA1 mutant (MK3941). PD-1 antibody combined with IL-2 treatment of mouse 3D-TSCs for 6 days. Cell viability of mouse 3D-TSCs were measured by MTT assay in SPC (658) and BRCA1 mutant (MK3941) tumor samples. n = 4. Error bars ± SEM, *p < 0.05; **p < 0.01; ***p < 0.001; ****p < 0.0001 (control versus αPD-1/αPD-L1/IL-2).

To test the immunotherapy efficacy of 3D-TSCs, we subsequently detected the response of primary mouse and human tumors to PD-1 and PD-L1 blockade in 3D-TSCs. The study used IgG antibodies as treatment controls. Cell viability was found to decrease after treatment with αPD-1 (Figure 5A) or αPD-L1 (Figure 5B) in liver cancer slices derived from a genetically engineered mouse model, except for three tumors (597-T1, T2 and T3), which were derived from the same mouse. Staining of antibodies to PD-L1 revealed that the expression levels of PD-L1 in these tumors ranged from 17-71% (Figure 5C and Figure S9A). These data suggests that expression of PD-L1 at a certain level, even at a level of approximately 17% of cells, could sensitize cancer cells to PD-1/PD-L1 blockage, whereas even if up to about 50% of cells express PD-L1 in some tumors, such as 597-T1-3 and 607, their sensitivity remained low. This observation highlights the necessity for drug-sensitive testing before clinical treatment.

Meanwhile, the response of PDTS derived from colon cancers to anti-PD1 or anti-PD-L1 treatment was also evaluated. These tumors expressed PD-L1 at low levels, ranging from 2%-11%, and were also less sensitive to PD-1/PD-L1 blockade than mouse tumors tested earlier (Figure 5D-F Figure S9B). Colon Pt 4 expressed PD-L1 at about 11% cells and displayed higher sensitivity than other cancers that exhibited PD-L1 at lower levels. Altogether, our data indicate that higher expression levels of PD-L1 in cancer cells, in general, render cancers higher sensitivity to drugs although with some exceptions.

Next, in order to determine if T cells could be activated in PDTS by the αPD-L1 and αPD-1 treatment, qRT-PCR analysis of T cell markers programmed cell death 1 (*PDCD1*), the cytolytic markers perforin-1 (*PRF1*), and granzyme B (*GZMB*) was performed in PDTS. The data revealed that although αPD-1 treatment increased expression of the *PDCD1* and αPD-L1 elevated expression of all three genes, none of the changes reached statistically significant level compared to the untreated controls based on student's *t*-test analysis (Figure 5G). It was previously indicated that IL-2 expanded tumor-infiltrating lymphocytes (TILs) and has remarkable efficacy in immunogenic tumors 40, we herein tested combinatory effects of αPD-1 and IL-2 in our 3D-TSC system (Figure 5H, I). The data indicate that combinatory treatment on 3D-TSCs derived from a mouse SPC (658) cancer elicited higher killing effect than the mono-treatment, yet the enhancement did not reach a statistically significant level (Figure 5H). We suspected this might be due to the fact that this 3D-TSC already respond to mono-treatment of αPD-1 so well, which might overshadow additional effect of IL-2. Therefore, we test another 3D-TSC derived from BRCA1 mutant (MK3941), which was insensitive to αPD-1, and the data revealed a significant statistically response of the combinatory treatment of αPD-1 or IL-2 alone (Figure 5I). These experiments suggest in principle that potential effects of various drug combinations could be tested using the 3D-TSC system.

### Real-time monitoring of 3D-TSCs response to chemotherapy and immunotherapy based on label-free fluorescence imaging techniques

**Figure 6:**
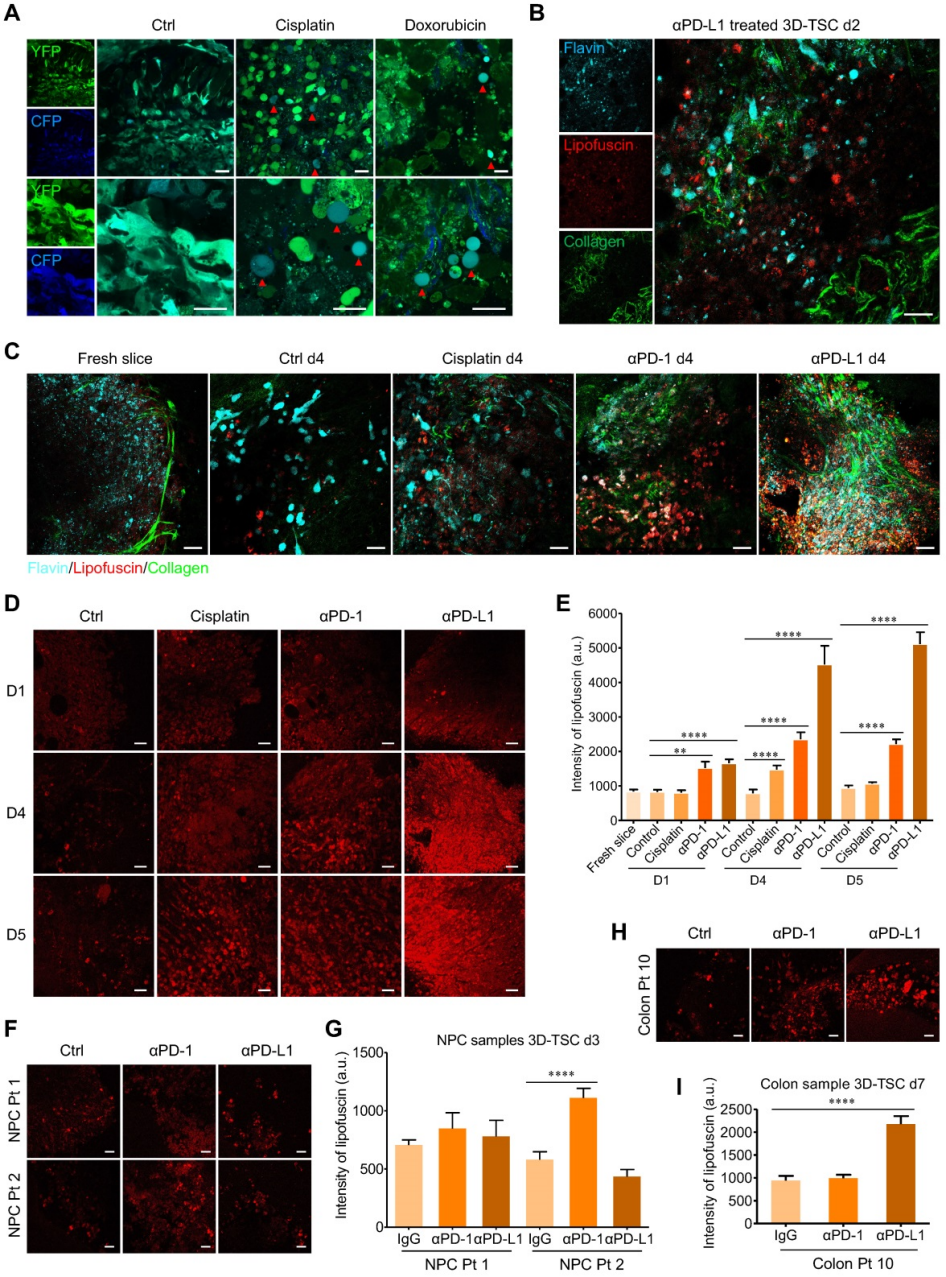
** Real time monitoring of 3D-TSCs response to chemotherapy and immunotherapy based on label-free fluorescence imaging techniques. A** Two-photon fluorescence microscope imaging of cell apoptosis in 3D-TSCs. MDA-MB-231-C3 tumor slices treated with 100 µM cisplatin or 10 µM doxorubicin for four days. Red arrows indicate apoptotic cells. Scale bar: 24 µm. **B** Two-photon autofluorescence imaging of collagen (λ_ex_ = 740 nm), lipofuscin (λ_ex_ = 1040 nm) and flavin (λ_ex_ = 890 nm) in the genetically engineered mouse tumor slices treated with 2.5 μg/mL αPD-L1 for two days. Scale bar: 24 µm. **C** Two-photon autofluorescence imaging of collagen, lipofuscin and flavin in the genetically engineered mouse tumor slices treated with 25 µM cisplatin, 2.5 μg/mL αPD-1 or 2.5 μg/mL αPD-L1 for four days. Scale bar: 24 µm. **D** Two-photon autofluorescence imaging of lipofuscin in the genetically engineered mouse tumor slices treated with indicated drugs for 0-5 days. Scale bar: 24 µm. **E** Evaluation of lipofuscin autofluorescence intensity in the genetically engineered mouse tumor slices treated with indicated drugs. n = 27. **F, G** Two-photon autofluorescence imaging of lipofuscin and evaluation of lipofuscin intensity in the human nasopharyngeal cancer slices treated with 10 μg/mL αPD-1 or 10 μg/mL αPD-L1 for three days. Scale bar: 24 µm. n = 12. **H, I** Two-photon autofluorescence imaging of lipofuscin in the human colon cancer slices (Colon Pt 10) treated with 10 μg/mL αPD-1 or 10 μg/mL αPD-L1 for seven days. Scale bar: 24 µm. n = 15. Error bars ± SEM, **p < 0.01; ****p < 0.0001 (control versus cisplatin/αPD-1/αPD-L1).

Compared to fluorescent dye labeling, label-free optical methods allow for the rapid, non-invasive, and time-course monitoring of tissue morphology and metabolic status, which have significant applicability in clinical and/or commercial endeavors 41. As a parallel validation, we confirmed that two-photon fluorescence imaging system could monitor drug-induced cell apoptosis in 3D-TSCs derived from MDA-MB-231-C3 tumor (Figure 6A). Two-photon microscopy of non-fluorogenic counterparts was then performed, based on the molecular imaging of collagen, flavins, and stress-induced lipofuscin in cells (Figure 6B). The 3D-TSCs treatment dynamics were assessed using a Nikon A1R Ti2-E multiphoton inverted microscope. With the exception of flavins as the indicators of oxidative metabolism in cells, we selectively excited lipofuscin's 600 nm red autofluorescence at 1040 nm (spectrum validation in Figure S10A). After treating with 25 µM cisplatin, 2.5 μg/mL αPD-1, or 2.5 μg/mL αPD-L1, a significantly increased in lipofuscin fluorescence intensity was observed in mouse tumor samples within 4 days using 3D-TSC (Figure 6C and Figure S10B). Next, we conducted the PI staining (Figure 6D, E and Figure S11) and MTT assay (Figure 6F and Figure S10C, D) and confirmed the increased fluorescence induced by all three agents was associated with cell death. To further test the label-free system, mouse GFP positive tumor slices were prepared, in which we confirmed the increase of lipofuscin fluorescence intensity and the decreased number of GFP positive cells during the course of 3D-TSC treatment (Figure S12). In a quick demonstration on PDTS samples (Figure 6G, H), lipofuscin fluorescence intensity was also showed significantly increased in PDTSs of colon cancer after treating with 10 μg/mL αPD-L1 for 7 days. For the assay of nasopharyngeal cancer, PDTSs from two patients presented different responses to ICB therapy. A slight increase of lipofuscin fluorescence intensity was found in αPD1/αPD-L1 treated PDTSs from patient 1 (Colon Pt 11). In contrast, there is a significant change of lipofuscin fluorescence for PDTSs from patient 2 (Colon Pt 12) after treating with 10 μg/mL αPD1 for 3 days. These results suggest that lipofuscin fluorescence could serve as a valuable optical reporter for label-free real-time monitoring of drug response in 3D-TSCs, with possible translatability to clinical practice.

## Discussion

In the present study, we have established a 3D-TSC platform that accurately preserves cell repertoire and immune components of the original tumor. After establishing an approach that allows time-course and quantitative assessment of cell death, we demonstrated that the platform is capable for testing individualized drug responses and immune checkpoint blockade therapy for surgically removed cancers. Compared to other *ex vivo* system, such as PDOs, which is promising in predicting anti-cancer drug sensitivity 12, 3D-TSC imparts several major advantages: 1) it contains stromal cells, immune cells, and cancer cells; 2) all resected cancer samples can be used for drug evaluation; and 3) it is a rapid and low-cost *ex vivo* system. In fact, the evaluation process could be complete within 4-7 days while PDOs require more than two weeks. These features make 3D-TSC a physiologically relevant model in the field of precision oncology for selecting the optimal and most potent drugs for each cancer patient. The culture conditions of our 3D-TSCs preserved important features of the original tumor, such as cancer heterogeneity and architecture, immune components, and gene expression. With the assistance of 3D-TSCs, drug efficacy was evaluated using human breast cancer and colon cancer cells within 4-7 days (Figure 4). The breast cancer samples tested were found to be more sensitive than the colon cancer samples. This may be attributed to the cell lines used for the primary selection of these drugs. For example, the MDA-MB-231 cells are already quite resistant to anti-cancer drugs, as previously reported 42-44. Indeed, our primary selection yielded much fewer drugs that were effective for MDA-MB-231-C3 cells than for Hct116-C3 cells (34 vs 88 drugs). Meanwhile, there are variations in drug response between PDTS especially Colon Pt 3, primarily because of differential growth rates among different slices due to the spatial heterogeneity of tumor and varying nutrient availability. In addition, both human and mouse tumor 3D-TSCs exhibit cytotoxicity responses to PD-1/PD-L1 checkpoint blockade within 7 days after the surgery (Figure 5). Given the current immune therapies are very costly, and the expression levels of PD-L1, sometime do not faithfully predict the efficacy of the therapy 45, 3D-TSC model might be useful for testing sensitivity of immune checkpoint blockade mediated by PD-1/PD-L1. Tumor slices can be prepared from tumor samples derived from either surgery or biopsy. All NPC samples were obtained by biopsy. Due to the limited quantity of tumors from biopsy, we usually prepare no more than 30 slices from each tumor for drug test with 3 slices for each drug (Figure 4C-H). For other types of tumors, such as breast and colon, most samples were obtained by surgical resection, and more tumor slice could be prepared. Our 3D-TSC platform could finish drug test within 7 days after surgery or biopsy, but when the patients could be treated is upon the decision of the doctor.

Traditionally, evaluating drug responses relied on destructive fixation and toxic dye labeling that was destructive and toxic to cells, respectively. Consequently, valuable cytomic and metabolic information are discarded. To overcome the challenges associated with the lack of effective predictive biomarkers or reporters for drug discovery, this study established cancer cells that express FRET-based caspase reporter C3 as a reporter to screen 166 anti-cancer drugs to identify high efficacy drugs. This was achieved by both inhibiting cell growth and induction of apoptosis. This approach should be suitable for large-scale of drug screening to narrow down potential candidates for further analysis.

With the exception of transgenic fluorescent reporters, intrinsic metabolic fluorophores could also provide valuable alternatives. Label-free imaging technologies have made remarkable advances in recent years, such as visualization of the tumor microenvironment 46, membrane potential 47, iron-bound transferrin in unlabeled intact breast cancer cells and tumor xenografts 48, cell-cycle status 49, and cell metabolic response 50-52. Label-free imaging has also been used to image patient-derived organoid cultures including colon cancer 53, head and neck cancer 54, and pancreatic cancer cultures 55. To our knowledge, this is the first report of label-free fluorescence imaging technology used for time-course imaging of 3D-TSCs. Our results indicate the potential of label-free fluorescence imaging in the visualization of 3D-TSC pharmacodynamics. Notably, lipofuscin red fluorescence has been discovered as a novel label-free optical indicator to assess drug efficacy in clinical samples, which paves a new route for personalized drug discovery that does not require dyes or sacrificing samples.

In summary, 3D-TSC demonstrates its strong potential for testing compounds or antibodies directly on the patient-derived tumor tissue in relatively short time not only for targeted chemotherapy but also for immunotherapy. Armed with a transgenic reporter system and label-free metabolic imaging techniques, we believe that 3D-TSC could accelerate drug discovery, improve cancer treatment by providing accurate clinical guidance, and, therefore, might improve personalized therapeutic options for individual patients when standard clinical options have been exhausted.

## Methods

### Cell culture and drug treatment

The immortalized mouse Brca1-WT epithelial cell line B477 was derived from the mammary gland of Brca1-WT mice (*Brca1*+/+;*p53*+/-) as previously reported 56. Cancer cell lines MDA-MB-231, HepG2, HCT116, PANC-1, and A549 were purchased from the American Type Culture Collection (ATCC, Manassas, VA, USA), were cultured in Dulbecco's modified Eagle's medium (DMEM, Life Technologies) supplemented with 10% fetal bovine serum (FBS, Life Technologies) and 1% antibiotics (penicillin and streptomycin, Life Technologies) in 5% CO_2_, 95% air at 37 °C in humidified incubator and were then collected at the designated time-points for various analyses.

The anti-cancer drugs were purchased from Selleck Chemicals (Houston, USA), Sigma-Aldrich (Shanghai, China) and J&K Scientific Ltd (Beijing, China). All drugs in drug library for drug screening were first stored in DMSO at the concentration of 10 mM and then diluted with culture medium and used at working concentration of 20 μM.

### Mice and tumor preparation

All mouse strains were maintained in condition with a 12 light/12 dark cycle and temperatures of 18-23 °C with 40-60% humidity. Mouse xenograft tumors labeled with Sensor C3 were established by subcutaneous injection of C3-labeled cancer cells (5×10^5^) were injected into each flank site of nude mouse. The immortalized mouse *Brca1*-WT epithelial cell line B477 was implanted into the right mammary fat pads of nude mice. All animal experiments were conducted under the supervision of the University of Macau Animal Ethics Committee (UMAEC-050-2015).

### Three-dimensional tumor slice cultures (3D-TSCs) and drug treatment

3D-TSC was prepared from tumors developed in the genetically engineered mouse models, xenograft tumors, or human primary tumors as previous described 57, 58 with the following modifications that are critical for maintaining viability of the slice in *ex vivo* condition. Tumors were collected and placed into cold media (DMEM media with 10% FBS and 1% Penicillin/Streptomycin (Gibco). Thick tissue slices (300 μm) were prepared under sterile conditions using a vibratome, Leica VT1200 S (Leica Biosystems Nussloch GmbH, Germany) within 2-6 h after surgery. Mix ice-cold A (Rat Collagen I), B (10 X Ham's F-12), and C (sterile reconstitution buffer, 2.2g NaHCO3 in 100ml of 0.05N NaOH and 200mM HEPES) at a ratio 8:1:1. Pour 100 μL of reconstituted collagen solution into each 12 mm diameter Millicell insert (Millipore, PIHP01250) in a 37 °C incubator. Leave inserts in tissue culture incubator for 20-30 min until collagen solidifies completely. Slices were then carefully placed on 0.4-mm pore size membrane culture inserts precoated with collagen gel mixture in 24-well plates. After solidifying of the top layer tissue-containing gel, add 400 μL culture medium into the outer dish in the hood. Slice culture medium is composed of Ham's F12, 20% fetal bovine serum and 50 μg/ml gentamicin. Later, slices were cultivated in a humidified incubator at 5% CO_2_ and 37 °C. Medium was changed every 4 d thereafter. The slices at different time-point were taken out for imaging analysis with a Leica M165FC fluorescent stereo microscope.

To elucidate the immune resistance mechanism of immune checkpoint surface receptor programmed cell death protein-1 (PD-1) and programmed death-ligand 1 (PD-L1), mouse tumor slices were treated with IL-2 (Peprotech, 500 IU/ml), control mouse IgG (EMD, 10 μg/mL), anti-mouse-CD279 (αPD-1, Biolegend, 10 μg/mL) or anti-mouse-CD274 (αPD-L1, Biolegend, 10 μg/mL) as indicated. For PDTS, all human cancer samples were derived from Kiang Wu Hospital, Macau. Tumor slices were treated with control human IgG4 (Sigma, 10 μg/mL) or Pembrolizumab (αPD-1, 10 μg/mL), control human IgG1 or Durvalumab (αPD-L1, 10 μg/mL) as indicated.

### Histochemical and histological analysis

3-(4,5-Dimethylthiazol-2-yl)-2,5-diphenyltetrazolium bromide (MTT) is a tetrazolium salt that is commonly used to quantitatively measure living cells. The amount of MTT formazan formed in mitochondrial is directly proportional to the number of living cells^58^ . Propidium Iodide (PI) is used to identify dead cells, as it is membrane impermeant and generally excluded from viable cells, but penetrates dead or damaged cells and binds to DNA and RNA by intercalating between the bases 59. We used above two reagents to detect cell death in 3D-TSCs with non-fluogenetic signal. After drug treatments, 3D-TSCs were washed twice with cold PBS followed by the addition of PI (100 µg/mL), and incubated for 3h at room temperature in the dark. 3D-TSCs were washed with 1×PBS buffer. Images were obtained using a fluorescence stereomicroscope (Leica, M165FC, Germany). 3D-TSCs that stained with PI+ pattern were considered to be in late apoptosis or necrosis. For MTT, 200 µL of serum-free media mixed with 200 µL of 50 μg/mL MTT solution were added into each well in 24 well plate, followed by incubating the plate at 37 °C for 3 h. 3D-TSC was washed with PBS buffer for three times. 800 µL isopropanol was then added into each well and the plate was shaken on an orbital shaker for 30 minutes to solubilize the formed formazan. The optical density at 570 nm was measured using a plate reader (Perkin Elmer Victor X3, Waltham, MA, USA).

Tumor slices derived from genetically engineered *Brca1^Co/Co^;MMTV-Cre* mouse 35 were fixed with 10% formalin for overnight at room temperature and embedded in paraffin for immunohistochemistry stain or immunofluorescence analysis as indicated (Table S7). Immuno-stained slides were analyzed using the IHC toolbox plugin of ImageJ software to identify proportion of the brown color of H-DAB stained images and separated from Harris hematoxylin 60. Quantification of cell fluorescence in immuno-stained slides were performed by calculating area of all cells of interest and separated from fluorescence of DAPI using the ImageJ software. For each sample, at least 5 representative fields were captured for analysis.

### qPCR and RNA-seq

Total RNA was extracted using TRIzol reagent (Invitrogen), and cDNA synthesis was carried out using Reverse Transcriptase enzyme (Qiangen). RT-qPCR reactions were performed on a QuantStudio 7 Flex instrument (Applied Biosystems) using FastStart SYBR Green Master Kit (Roche, Germany) according to manufacturer's protocol (Table S8). The relative expression data were measured and normalized to inner control genes 18s. Primers for qRT-PCR analysis of human immune genes were listed in Table S9.

For RNA sequencing, to minimize effects of intra-tumor heterogeneity on slice culture, mouse slices derived from center of tumors were cut using a vibratome and shipped to Novogene Corporation (Beijing, China) for library construction and sequencing. Transcripts per million (TPM) is used to estimate transcript or gene expression levels. Many studies apply TPM to represent RNA expression value and TPM becomes the most widely used method for RNA-Seq normalization 61. Besides, TPM is the best method for RNA-Seq normalization compared to the other methods 62. FPKM (reads per kilobase of transcript per million rads mapped) can be converted to TPM by a easily formula: TPM = RPKM / (sum of RPKM) ×10^6^ 63. Gene Set Variation Analysis (GSVA) package is used to measure gene set enrichment (GSE) in RNA-Seq data. Single sample GSEA (ssGSEA) method in GSVA package, an extension of Gene Set Enrichment Analysis, calculates a gene set enrichment score for each pairing of a sample and gene set. Generally, each ssGSEA sore represents the level of the gene set is up- or down-regulated within a sample 64.

### Measurement of cell viability in 3D-TSCs

To determine the cell viability of tumor slices during 3D culture, tumor slices with same size were seeded on collagen gel-coated inserts in 24-well plates and grown for 10 days. 3D-TSCs were collected and enzymatically dissociated into single cells at 37 °C using Digestion I (10 mg/mL insulin, 10 mg/mL hydrocortisone, 100 ug/mL EGF, 1.2 mg/mL collagenase III, 0.02 mg/mL hyaluronidase in DMEM/Hams F12) for 2 h and followed by digestion II (5 mg/mL dispase II, 0.1 mg/mL DNAase I, 20 mM HEPES in DMEM/Hams F12) for 5 min. Cells were filtered through a 0.40-μm strainer and pelleted, resuspended in PBS buffer. Cells were mixed with an equal volume of trypan blue and cell viability was counted using a Nexcelom Bioscience Cellometer Auto 2000.

### Fluorescence microscopic imaging and multiphoton fluorescence microscopy

Widefield imaging of live cancer cells expressing C3 was observed by a Nikon Eclipse Ti-E fluorescent microscope equipped with 10× 0.3 NA objective lens. The emission images of YFP (525 nm) and CFP (480 nm) were recorded. For time lapse imaging of cells, dishes were placed in a humidified cell-culture incubator and continuously supplied with 5% CO_2_ at 37 °C on a Nikon Eclipse Ti-E fluorescent microscope. In the merged FRET images, live cells appeared in cyan color while apoptotic cells appeared in blue color. Drug efficacy was quantified by measuring both apoptotic cells and number of survived cells using R Studio software. FRET signal of tumor slices was measured on the stage of a Leica M165FC fluorescent stereo microscope. The ImageJ 1.48v software (National Institutes of Health, USA) was used to quantify FRET ratio (YFP/CFP ratio).

Multiphoton fluorescence imaging of tumor slices was observed by a Nikon A1R Ti2-E multiphoton inverted microscope. Slices (200 μm) were placed on culture inserts precoated with 100 μL collagen gel mixture in 24-well plates and were cultivated in a humidified incubator at 5% CO_2_ and 37 °C. Using Spectra-Physics' femtosecond laser InSight X3, we excited the CFP of tumor cells at 860 nm and detect the blue fluorescence at 415-485 nm spectral range. The 430 nm second harmonic generation signal was blocked by a 450 nm long-pass filter in that detection channel. The YFP FRET signal from CFP was simultaneously detected at another channel with 506-593 nm detection range. For the label-free imaging of treatment response in tumor microenvironment, tumor slices were treated with cisplatin (25 µM), anti-PD1 (αPD-1, 2.5 μg/mL) or anti-PD-L1 (αPD-L1, 2.5 μg/mL). The multiphoton fluorescence imaging was performed before and at the 1^st^, 2^nd^, 4^th^, and 5^th^ days post treatment. Metabolic autofluorescence intensities of cancer cells are assessed and analyzed. The collagen distribution was revealed by the 740 nm and 1040 nm excited second harmonic generation (SHG) contrasts. The flavin autofluorescence of slices was captured at an excitation wavelength of 890 nm and lipofuscin at an excitation wavelength of 1040 nm.

### Statistical analysis

All statistical data were analyzed using GraphPad Prism 8.2.1 software and presented as the means ± s.e.m. To calculate the percentage of cell apoptosis, we measured the FRET rate (YFP/CFP rate) of cell and 3D-TSCs from three independent experiments. All data were analyzed with Student's *t*-test, and the *p* value <0.05 was considered statistically significant.

## Supplementary Material

Supplementary figures and methods.

Supplementary table 1.

Supplementary table 2.

Supplementary table 3.

Supplementary table 4.

Supplementary table 5.

Supplementary tables 6-8.

## Abbreviations

231C3: MDA-MB-231C3
3D-CTS: 3-dimensional cultured tumor slice
C3: Sensor C3
FBS: fetal bovine serum
ATCC: American Type Culture Collection
DOX: doxorubicin
FRET: fluorescence resonance energy transfer
IL-2: interleukin-2
MTT: 3-(4,5-dimethylthiazol-2-yl)-2,5-diphenyltetrazolium bromide
PDO: patient-derived organoids
PDTS: patient derived tumor slice
PD-1: programmed cell death protein-1
PD-L1: programmed death-ligand 1
PI: Propidium Iodide
SPC: *Smad4^co/co^;Pten^co/co^;Alb-Cre*
