# Supplementary material for: Accelerating precision anti-cancer therapy by time-lapse and label-free 3D tumor slice culture platform
Source: Theranostics. 2021 Sep 13;11(19):9415–30. doi: 10.7150/thno.59533 (PMC8490519; doi:10.7150/thno.59533)
Supplement: Supplementary file 1 — Supplementary figures and methods. [file thnov11p9415s1.pdf]

Supplementary Information

Accelerating precision anti-cancer therapy by time-lapse and label-free 3D tumor slice culture platform

Fuqiang Xing[1,2,3,6], Yu-Cheng Liu[1], Shigao Huang[1], Xueying Lyu[1,2], Sek Man Su[1,2], Un In Chan[1,2], Pei-Chun Wu[1], Yinghan Yan[1], Nana Ai[4], Jianjie Li[1,2], Ming Zhao[1,2], Barani Kumar Rajendran[1,2], Jianlin Liu[1,2], Fangyuan Shao[1,2], Heng Sun[1,2,3], Tak Kan Choi[1,2], Wenli Zhu[5], Guanghui Luo[5], Shuiming Liu5, De Li Xu5, Kin Long Chan5, Qi Zhao[1,2,3], Kai Miao[1,2,3], Kathy Qian Luo[1,2,3], Wei Ge[3,4], Xiaoling Xu[1,2,3], Guanyu Wang[6⊠], Tzu-Ming Liu[1,2,3⊠], Chu-Xia Deng[1,2,3⊠]

1. Cancer Center, Faculty of Health Sciences, University of Macau, Macau SAR, China.
2. Centre for Precision Medicine Research and Training, Faculty of health Sciences, University of Macau, Macau SAR, China.
3. MOE Frontier Science Centre for Precision Oncology, University of Macau, Macau SAR, China.
4. Centre of Reproduction, Development and Aging, Faculty of Health Sciences, University of Macau, Macau SAR, China.
5. Kiang Wu Hospital, Macau SAR, China.
6. Department of Biology, School of Life Sciences & Guangdong Provincial Key Laboratory of Computational Science and Material Design, Southern University of Science and Technology, Shenzhen, China.

⊠ Corresponding author: Guanyu Wang (wanggy@sustech.edu.cn), Tzu-Ming Liu (tmliu@um.edu.mo) and Chu-Xia Deng (cxdeng@um.edu.mo)

Supplementary Figures and Figure legends

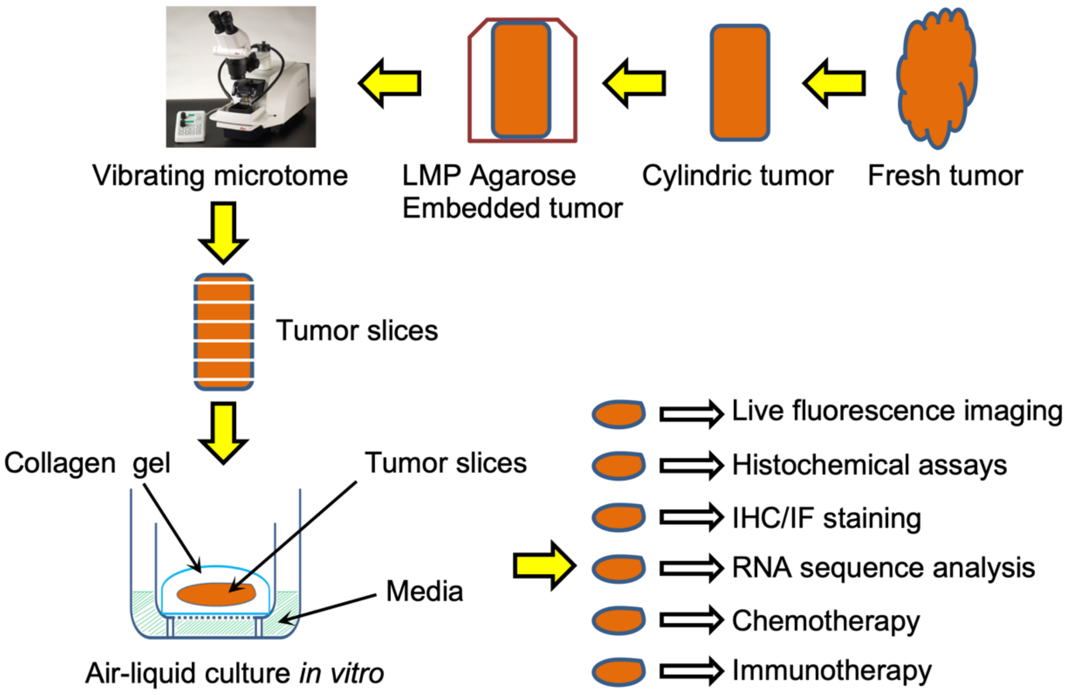

Figure S1. The procedure flowchart for tumor slices sectioning and 3D-TSCs. Tumor tissue is transported from the operating room to the laboratory and cut into 300 μm slices in D-PBS solution using a vibratome. The slices are transferred to culture medium and then carefully placed on membrane inserts in 24-well plates to create an air-liquid interface.

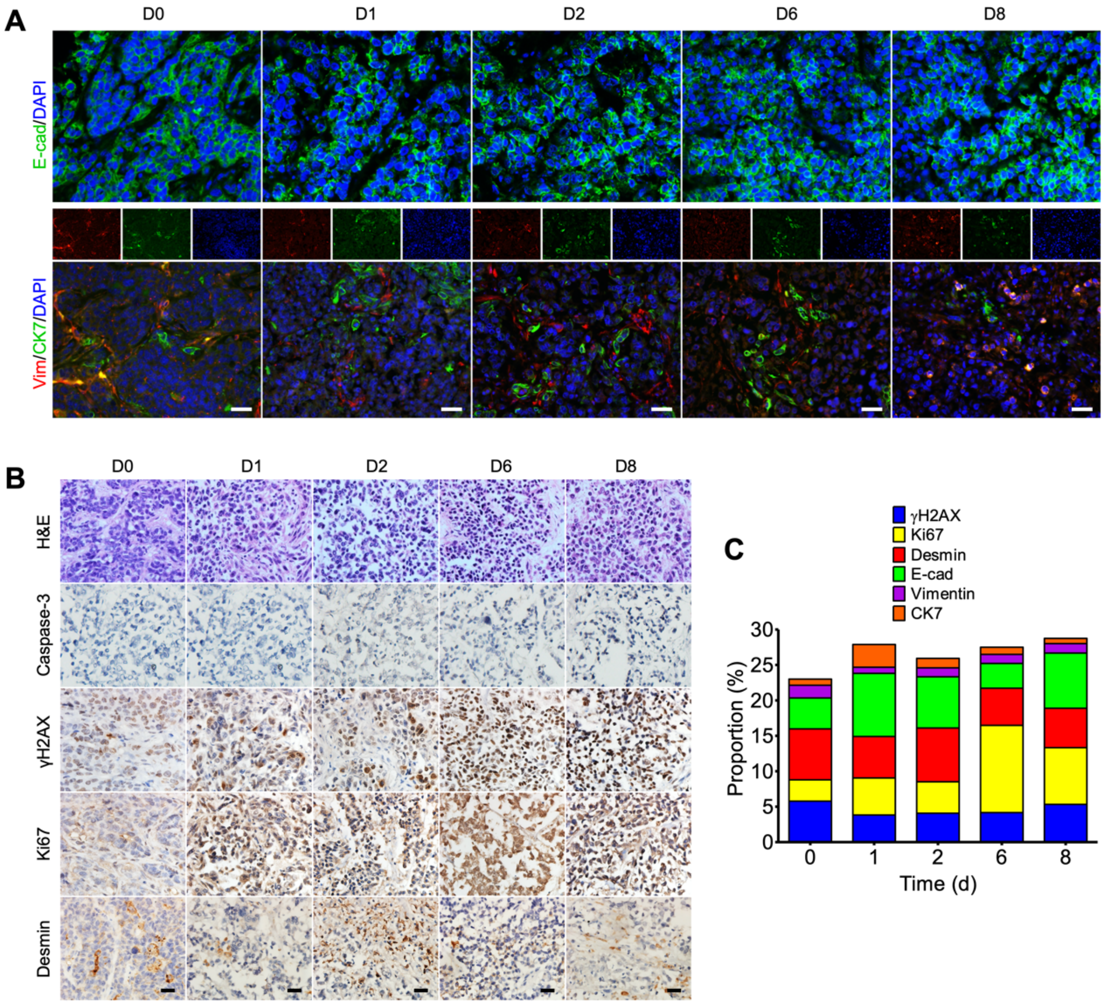

Figure S2. Histological analysis of biomarkers in 3D-TSCs derived from genetically engineered mouse tumor. A, B Histological analysis of biomarkers in 3D-TSCs derived from genetically engineered *Brca1*$^{Co/Co}$*;MMTV-Cre* mouse. Blue is nuclear counterstain by Hematoxylin, and brown staining is positive protein by DAB. γH2AX, marker for DNA double-strand breaks; Desmin regulates sarcomere architecture; E-cadherin, a calcium-dependent cell-cell adhesion molecule; Vimentin, maintain cellular integrity and anchor the position of the organelles in the cytosol; CK7, tumor parenchyma. Scale bar: 100 μm; **C** Quantitation of biomarkers in 3D-TSCs for panels A,B. Caspase-3 is not activated, therefore, there is no counting for it. 5 digital images of antibody-stained tissue slides were captured for calculating the mean in each sample.

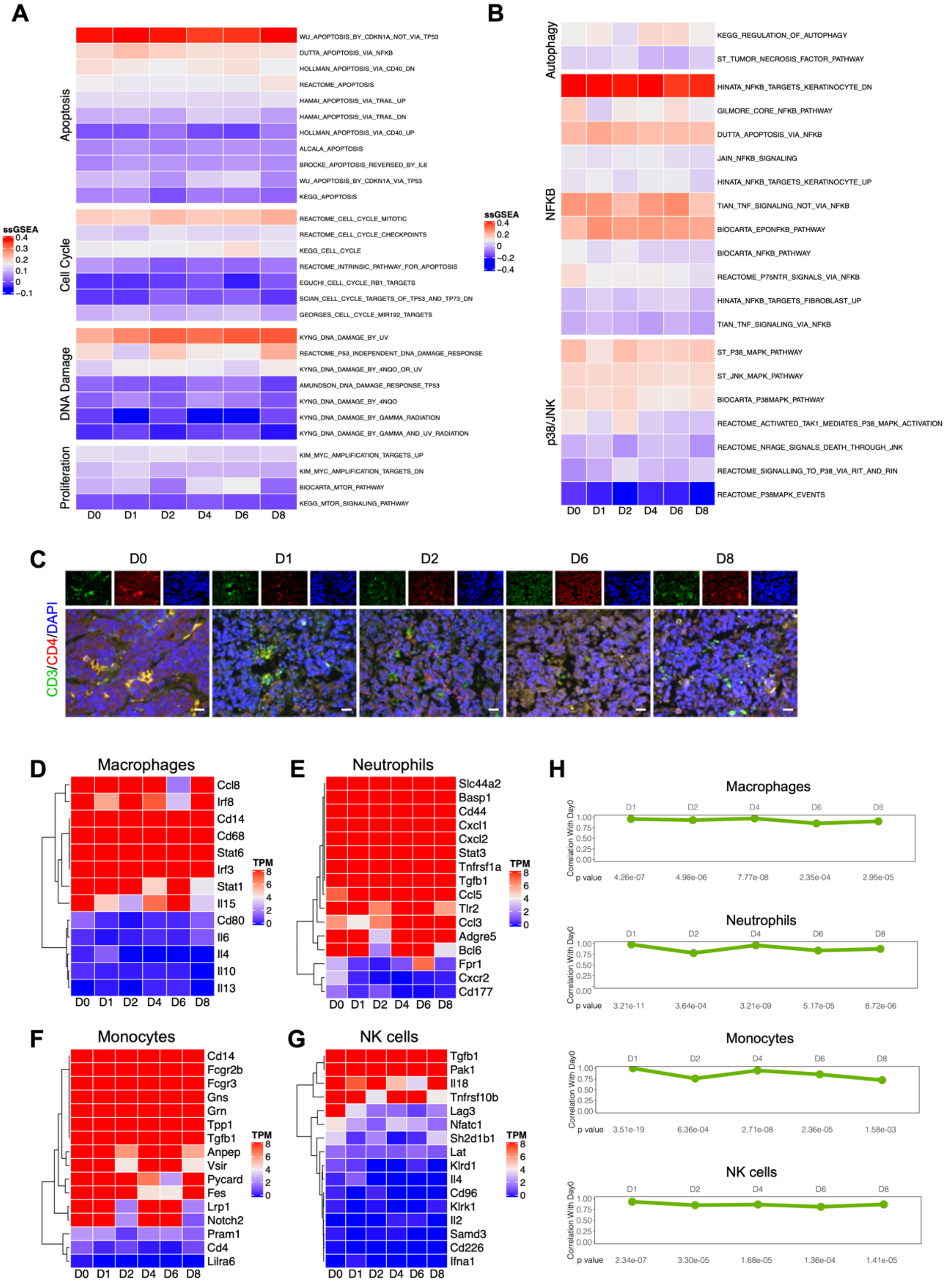

Figure S3. Histological and RNA seq analysis of marker gene expression in 3D-TSCs derived from genetically engineered mouse model. A, B Expression of pathway genes by RNA sequence analysis in 3D-TSCs derived from genetically engineered mouse. Single sample GSEA (ssGSEA) sore represents the level of the gene

set is up- or down-regulated within a sample; transcripts per million (TPM) is used to estimate transcript or gene expression levels; and expression of several pathways (including cell cycle, mismatch repair, ECM receptor interaction, Ubiquitin mediated proteolysis, and regulation of actin cytoskeleton) were analyzed by Pearson correlation coefficient for calculating the correlation. **C** Analysis of immune marker gene expression of 3D-TSCs derived from *Brca1*$^{Co/Co}$*;MMTV-Cre* mice. CD3/CD4: T lymphocytes; Scale bar: 100 μm. **D-G** RNA sequence analysis of immune marker gene expression for macrophages, neutrophils, monocytes, and NK cells. Transcripts per million (TPM) is used to estimate transcript or gene expression levels. **H** Comparison of the gene expression level by calculating the Pearson correlation coefficient for macrophages, neutrophils, monocytes, and NK cells from D1 to D8 with D0.

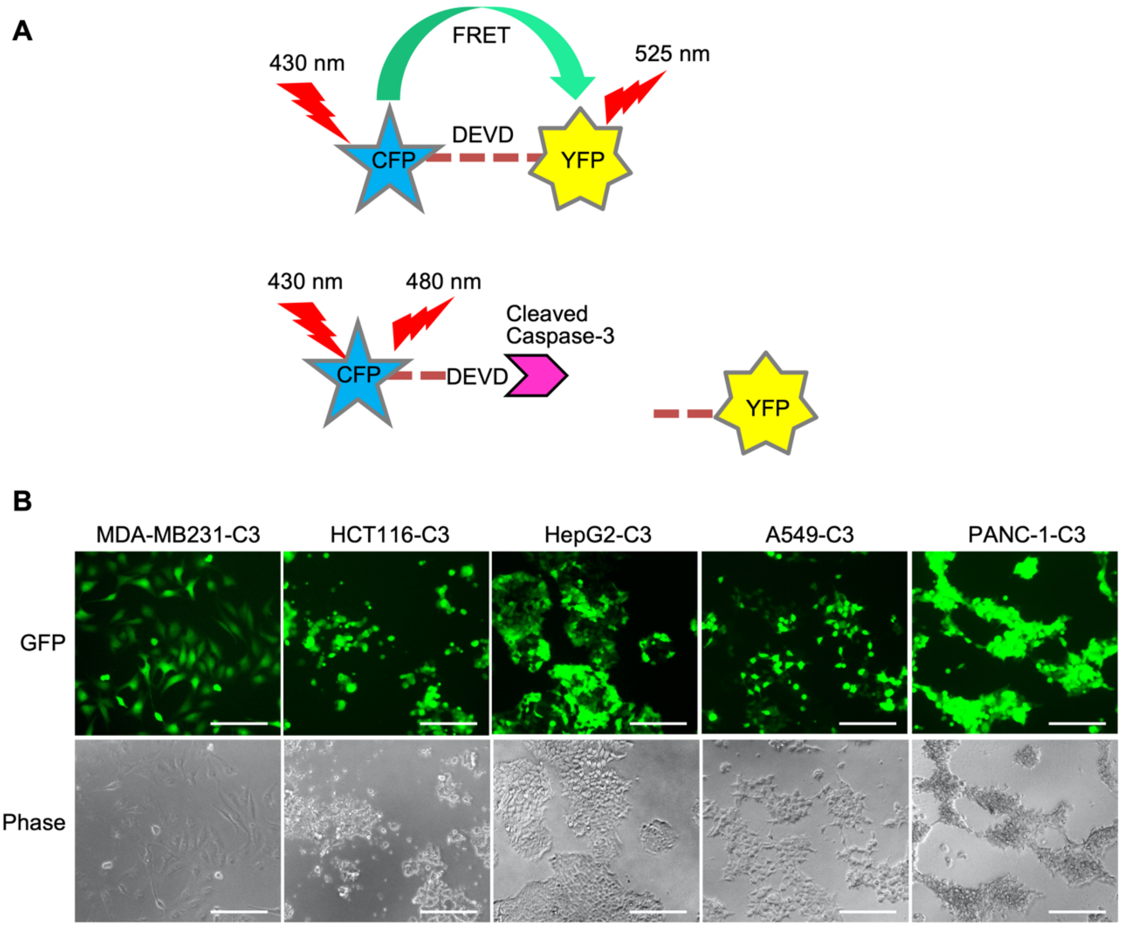

Figure S4. FRET-based biosensor for the detection of drug induced cancer cell apoptosis *in vitro*. **A** Principle of caspase-3 reporter Sensor C3. **B** Images of established

cancer cells expressing caspase 3 reporter Sensor C3. GFP images were captured with fluorescent microscopy using an EVOS® FL Cell Imaging System (Thermo Fisher Scientific). Scale bar: 50 μm.

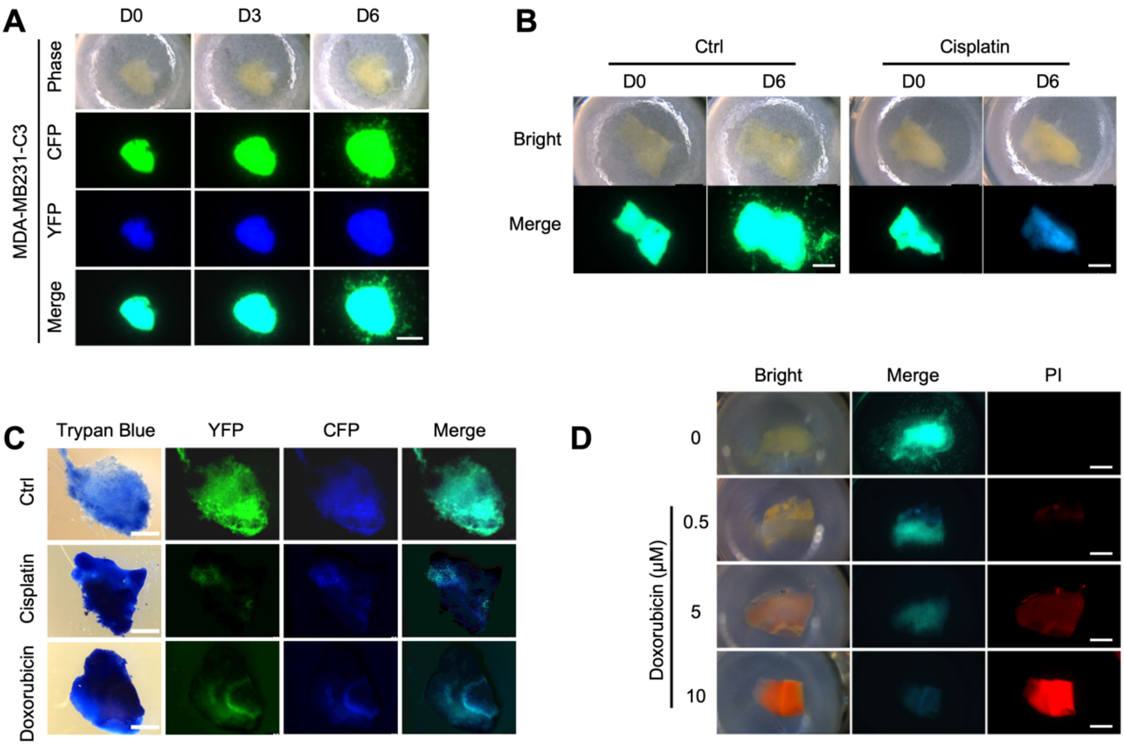

Figure S5. Validation of drug induced cell death in fluogenetic 3D-TSCs based on FRET-based biosensor C3. A Observation of tumor growth in 3D cultured MDA-MB-231-C3 tumor slices. Scale bar: 1 mm. **B** Imaging of tumor growth and CIS induced apoptosis; xenograft MDA-MB-231-C3 slices were treated with 100 μM CIS for 6d. **C** Trypan blue staining of cell death in MDA-MB-231-C3 tumor slices induced by 100 μM CIS or 10 μM DOX for five days. Scale bar: 1 mm. **D** Imaging of DOX induced cell death in MDA-MB-231-C3 slices by Propidium Iodide (PI) staining. Scale bar: 1 mm.

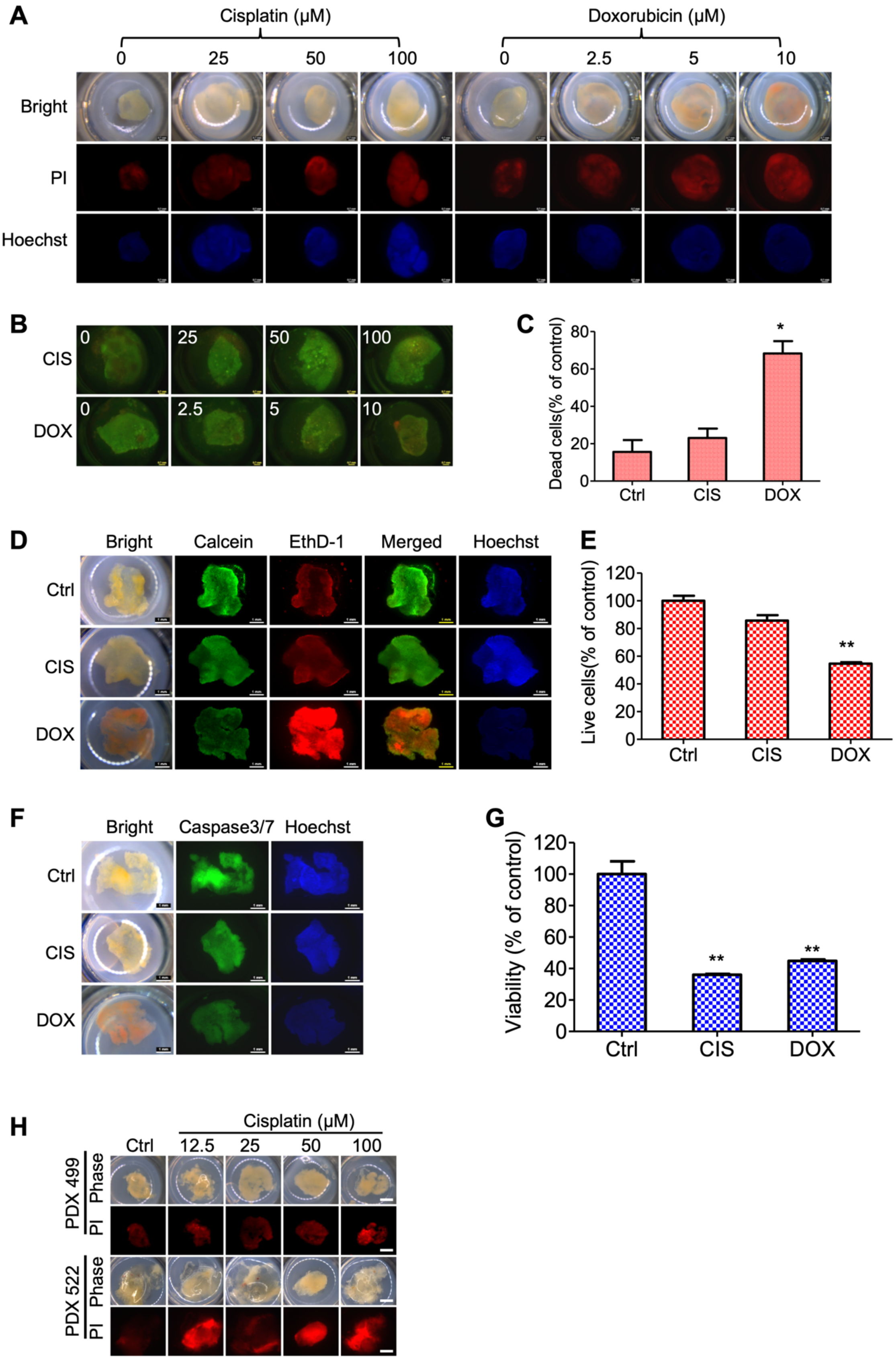

Figure S6. Drug response of non fluogenetic 3D-TSCs derived from genetically engineered mouse tumor to chemotherapy. **A** Imaging of CIS/DOX induced cell

death in 3D-TSCs derived from genetically engineered mouse model treated with CIS/DOX for 6 days by Propidium Iodide (PI) and Hoechst staining. **B, C** Detection of drug induced cell death in 3D-TSCs derived from genetically engineered mouse model treated with CIS/DOX for six days by LIVE/DEAD ® Viability/Cytotoxicity Kit. **D-G** Detection of drug induced cell death in 3D-TSCs derived from genetically engineered mouse model treated with CIS/DOX for four days by LIVE/DEAD ® Viability/Cytotoxicity Kit, Caspase-3/7 Green Detection Reagent and MTT; Scale bar: 1mm. n=3. **H** Imaging of PDX-Colon tumor slices treated with cisplatin for five days by PI staining. Scale bar: 1mm. n =3, error bars ± SEM, *p<0.05; **p<0.01 (control versus CIS/DOX).

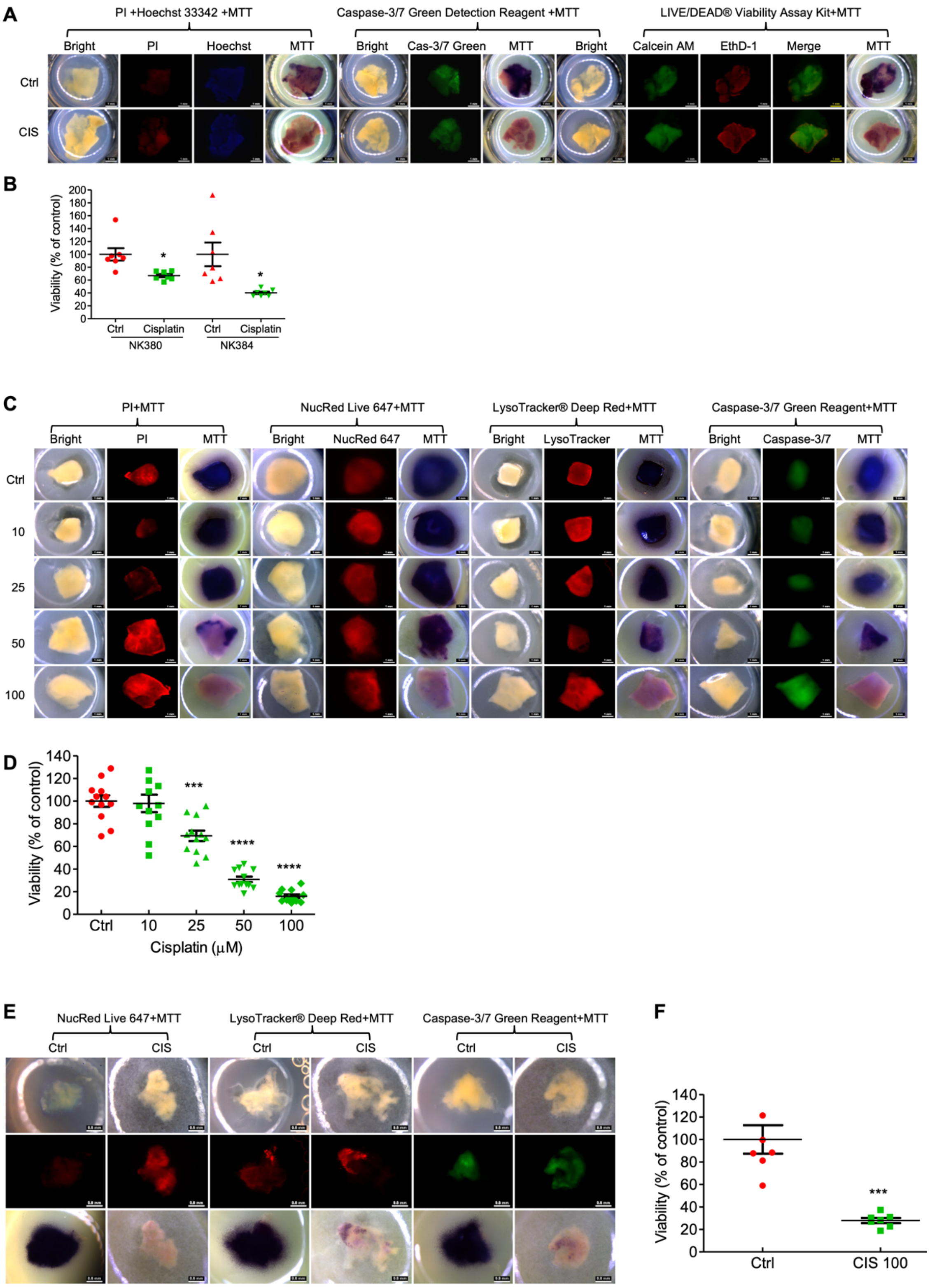

Figure S7. Detection of drug induced cell death in non fluogenetic 3D-TSCs derived from genetically engineered mouse model. A Detection of drug induced cell death in 3D-TSCs treated with CIS for six days by PI/Hoechst staining/Caspase-3/7 Green Reagent/LIVE/DEAD ® Viability/Cytotoxicity Kit and MTT; Scale bar: 1mm.

B MTT analysis of cell viability of 3D-TSCs treated with CIS for six days. n =7. **C, D** Detection of drug induced cell death in 3D-TSCs treated with CIS for six days by Propidium Iodide (PI)/NucRed Live 647/LysoTracker® Deep Red/Caspase-3/7 Green Reagent and and MTT assay; Scale bar: 1mm. n=12. **E, F** Detection of drug induced cell death in 3D-TSCs treated with CIS for six days by Caspase-3/7 Green Detection Reagent, LysoTracker probes, Live 647 ReadyProbes and MTT assay. Scale bar: 0.8mm. n =7, error bars ± SEM, **p*<0.05; ****p*<0.001; *****p*<0.0001 (control versus cisplatin).

A

Number of drugs that reach IC_{50} in the tested cell lines					
Either of the lines	231C3	HepG2-C3	Hct116-C3	PANC1-C3	A549-C3
94/166 (57%)	34/166 (20%)	32/166 (19%)	88/166 (53%)	37/166 (22%)	44/166 (27%)

Number of drugs that reach IC_{50} in one or more cell lines				
One cell line	Two cell lines	Three cell lines	Four cell lines	Five cell lines
38 (Hct116)<br>2 (A549)	9 (Hct116, A549)<br>4 (Hct116, 231)<br>1 (231, PANC1)<br>1 (Hct116, PANC1)<br>1 (Hct116, HepG2)	3 (Hct116, PANC1, A549)<br>3 (Hct116, PANC1, A549)<br>1 (Hct116, PANC1, 231)<br>1 (HepG2, PANC1, A549)<br>1 (A549, 231, Hct116)	4 (A549, Hct116, PANC1, HepG2)<br>1 (A549, 231, PANC1, Hct116)<br>1 (A549, 231, PANC1, 231)<br>1 (231, A549, PANC1, Hct116)	20

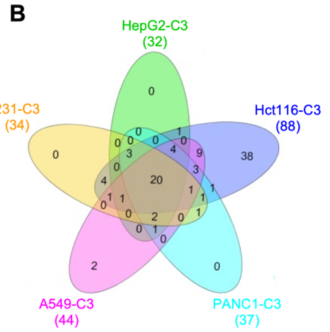

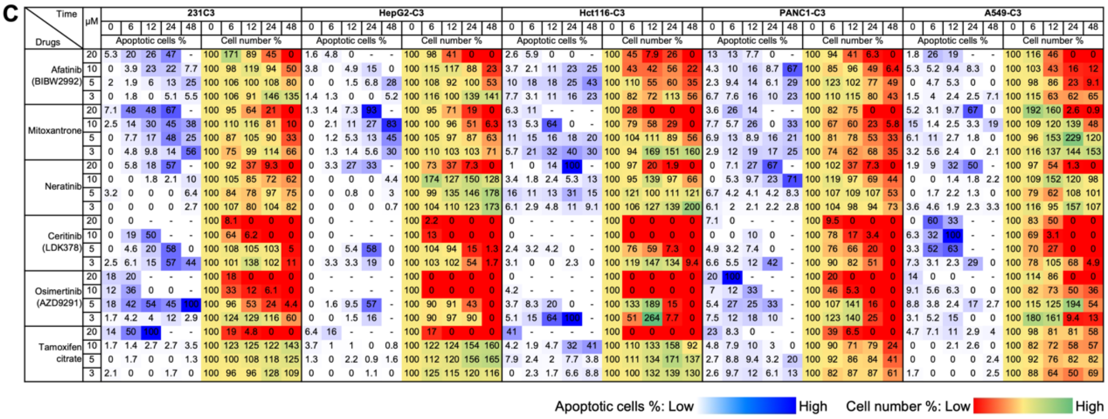

Figure S8. High throughput drug screening *in vitro*. A Summary of *in vitro* drug screening result in MDA-MB-231-C3, Hct116-C3, HepG2-C3, A549-C3 and PANC-1-C3 cell lines. **B** Venn diagram of drugs with high efficacy. **C** Heatmap of drug response with high efficacy, C3 cells were treated with indicated drugs at 5-20 μM for 0-48 hours. Apoptotic cells % is the percentage of apoptotic cells/cell numbers at each time point. Cell number % is the percentage of cell numbers (each time point)/cell numbers (0 hours). When cell numbers at the time point is “0”, (i.e. all cells have died as shown in most cases at 48 hours), “-” indicates no values.

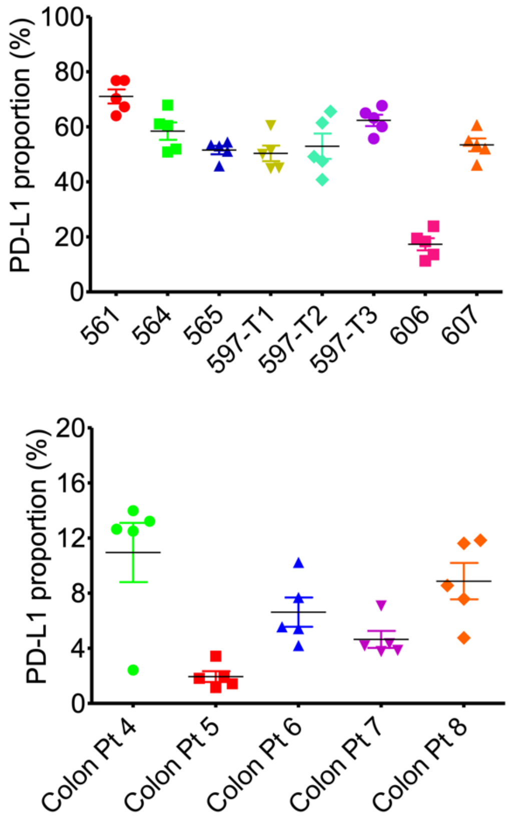

Figure S9. Quantification of original tumor PD-L1 IHC staining related with Figure 5. A Area quantitation of original tumor PD-L1 IHC in mouse surgical tumors. n = 5. **B** Area quantitation of original tumor PD-L1 IHC in human surgical tumors. n = 5. Error bars ± SEM.

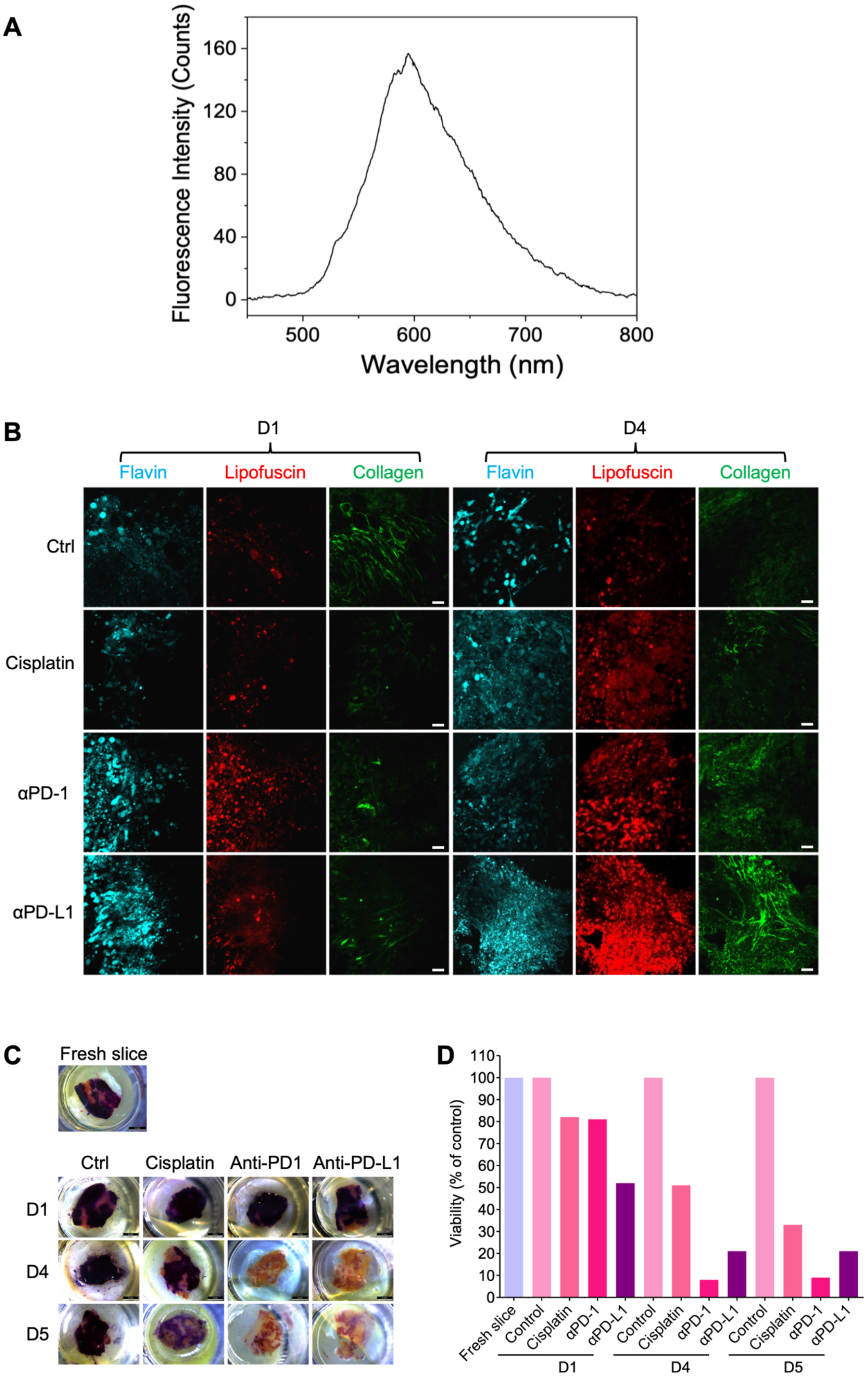
A
Fluorescence Intensity (Counts)
160
120
80
40
0
500
600
700
800
Wavelength (nm)
B
D1
D4
Flavin
Lipofuscin
Collagen
Flavin
Lipofuscin
Collagen
Ctrl
Cisplatin
αPD-1
αPD-L1
C
Fresh slice
Ctrl
Cisplatin
Anti-PD1
Anti-PD-L1
D1
D4
D5
D
Viability (% of control)
110
100
90
80
70
60
50
40
30
20
10
0
Fresh slice
Control
Cisplatin
αPD-1
αPD-L1
Control
Cisplatin
αPD-1
αPD-L1
Control
Cisplatin
αPD-1
αPD-L1
D1
D4
D5

Figure S10. Validation of lipofuscin as a label-free reporter of cell death in mouse breast 3D-TSCs. **A** Fluorescence emission spectrum of lipofuscin. **B** Second harmonic generation imaging of collagen (green, ex.@740 nm), two-photon fluorescence imaging of flavin (cyan, ex.@890 nm) and lipofuscin fluorescence (red, ex.@1040 nm). Scale bar: 24 μm. **C** MTT assay of drug efficacy in 3D-TSCs treated with 25 μM cisplatin, 2.5 μg/mL αPD-1 or 2.5 μg/mL αPD-L1. Scale bar: 2 mm. **D** Evaluation of cell viability in the mouse breast tumor slices treated with cisplatin by MTT assay.

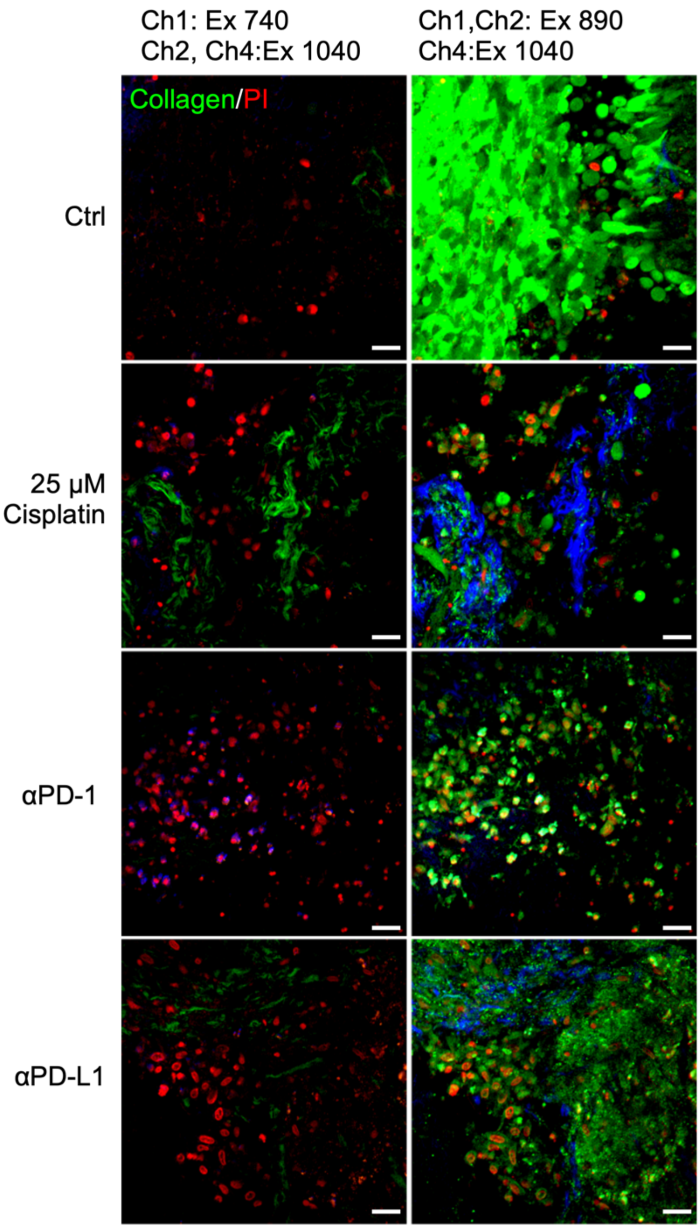

Figure S11. Confirmation of cell death by PI staining in mouse breast 3D-TSCs used for label-free imaging. (Left column) Combined two-photon fluorescence

imaging of NADH (blue, ex.@740nm), two-photon fluorescence imaging of propidium iodide (red, ex.@1040 nm), and second harmonic generation imaging of collagen (green, ex.@1040 nm). (Right column) Combined second harmonic generation imaging of collagen (blue, ex.@890 nm), two-photon fluorescence imaging of flavins (green, ex.@890 nm) and two-photon fluorescence imaging of propidium iodide (red, ex.@1040 nm). This set of images evaluate the cancer cell death by propidium iodide (PI) staining in genetically engineered mouse breast 3D-TSCs treated with 25 μM cisplatin, 2.5 μg/mL αPD-1 or 2.5 μg/mL αPD-L1. Scale bar: 24 μm.

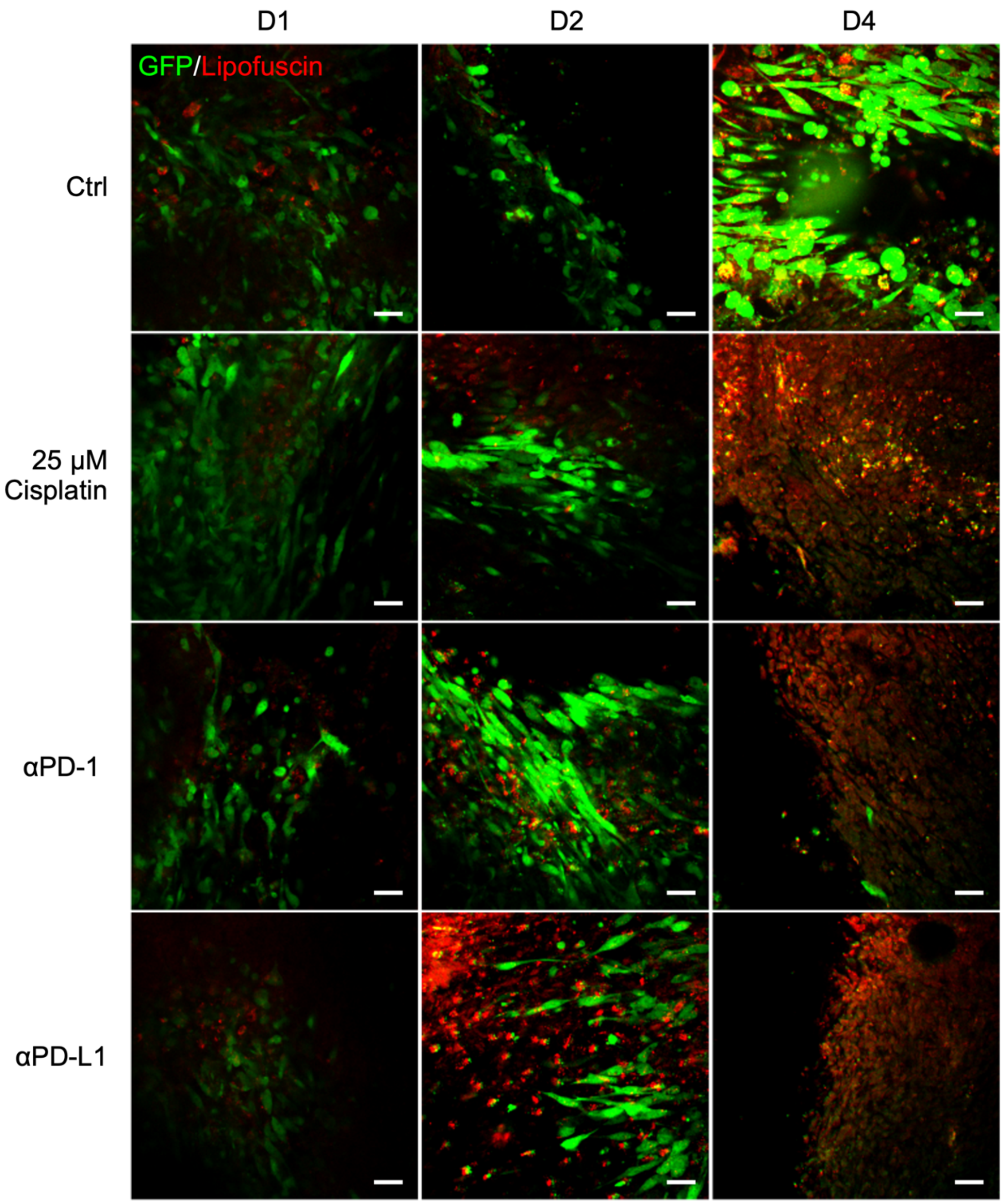

Figure S12. Validation of lipofuscin as a label-free reporter of cell death in mouse GFP+ 3D-TSCs. Combined two-photon fluorescence imaging of GFP (green, ex.@ 960 nm) and lipofuscin (red, ex.@1040 nm) in mouse GFP+ 3D-TSCs treated with 25 μM cisplatin, 2.5 μg/mL αPD-1 or 2.5 μg/mL αPD-L1. Scale bar: 24 μm.

Supplementary Methods

Generation of C3 labeled cancer cell lines.

Plasmid containing a caspase reporter C3 [1], a recombinant DNA unit that encodes a fusion protein CFP-DEVD-YFP, was purified using NucleoBond® Xtra kit. After linearization with *Stu*I, the C3 plasmid DNA was mixed with transfection reagent in a reagents/plasmid ratio of 2:1 (v/w). Adherent cells were seeded into a 12-well culture plate at 0.5-2 × 10^5/mL in 500 µl of growth medium per well without antibiotics. Transfection reagent Lipofectamine LTX was used to transfer into cancer cells and generated MDA-MB-231-C3, HepG2-C3, HCT116-C3, PANC-1-C3 and A549-C3 cells. Complexes were prepared as guided by the manufacturer's instructions. Reaction solutions were incubated for 20 min to form complexes. Cells were incubated at 37 °C in a CO_2 incubator for 6 h followed by replacing to normal medium. Fluorescence-activated cell sorting (FACS) of C3 labeled cells was performed to purify the stable cells.

Drug screening using FRET microscopy

For time lapse imaging, cultured cell dishes were placed in a humidified cell-culture incubator and continuously supplied with 5% CO_2 at 37 °C on a Nikon Eclipse Ti-E fluorescent microscope. Widefield imaging of live cancer cells expressing C3 was observed by a Nikon Eclipse Ti-E fluorescent microscope equipped with 10 × 0.3 NA objective lens. The emission images of YFP (525 nm) and CFP (480 nm) were recorded. In the merged FRET images, live cells appeared in cyan color while apoptotic cells appeared in blue color. The merged FRET images were analyzed to calculate the percentage of apoptotic cells using the following formula: % of apoptotic cells = Total number of blue cells/ Total number of green cells. Drug efficacy was quantified by measuring both apoptotic cells and number of survived cells using R Studio software. We used a cut off line for drugs that kill 50% cells and also trigger at least 10% apoptotic cells during this time-course. Because 20 µM is a relative high dose, if any drug that

does not meet these criteria, we concluded that the drug is not effective in inducing apoptosis for these cells and excluded them for further study.

Histochemical analysis

In addition to PI and MTT assay, we also tried other reagents to detect cell apoptosis in 3D-CTS. Hoechst 33342 is a cell-permeable chemical for staining DNA and nucleus, which can be used for specifically staining the nuclei of living or fixed cells, and reviewed by fluorescence microscopy. The LIVE/DEAD® Viability/Cytotoxicity Assay Kit is a two-color fluorescence cell viability assay that is based on the simultaneous determination of live and dead cells with two probes that measure recognized parameters of cell viability based on plasma membrane integrity and esterase activity [2]. The LysoTracker® Red DND-99 is fluorescent acidotropic probes for labeling and tracking acidic organelles in live cells [3]. CellEvent™ Caspase-3/7 Green Detection Reagent is a novel fluorogenic substrate for monitoring caspase-3 or -7 activation with live-cell fluorescence imaging [4]. NucRed Live 647 ReadyProbes reagent is a bright far-red cell-permeant nuclear stain for live or fixed cells [5]. We used above all reagents to detect cell death in 3D-CTS with non-fluogenetic signal. After drug treatments, the 3D-CTS were washed twice with cold PBS followed by the addition of above reagents and incubated for 3h at room temperature in the dark. The 3D-CTS were washed with 1×PBS buffer. Images were obtained using a fluorescence stereomicroscope (Leica, M165FC, Germany).

Measurement of fluorescence emission spectrum of lipofuscin

After drug treatment, obvious two-photon red autofluorescence ($\lambda ex = 1040$ nm) appear in the sectioning image of 3D tumor slices. To measure the fluorescence spectra, we zoom in the scanning range to a 6 x 6 μm region within cells carrying red autofluorescence. The emitted signals were epi-collected by the same objective and then reflected by an 865-nm edged dichroic beam splitter to an Andor Kymera 193i spectrograph equipped with an iDus 401 CCD. This spectrometer set is attached on the back-side port of the Nikon A1MP+ multiphoton microscope. In this integrated micro-

spectroscopy system, we could measure the spectra of certain imaging region on-demand. The spectra demonstrated an emission peak around 605 nm, which is characteristic features of lipofuscin in aging cells.

References

1. Luo KQ, Vivian CY, Pu Y, Chang DC. Application of the fluorescence resonance energy transfer method for studying the dynamics of caspase-3 activation during UV-induced apoptosis in living HeLa cells. Biochemical and biophysical research communications. 2001; 283: 1054-60.
2. Shang W, Liu Y, Kim E, Tsao C-Y, Payne GF, Bentley WE. Selective assembly and functionalization of miniaturized redox capacitor inside microdevices for microbial toxin and mammalian cell cytotoxicity analyses. Lab on a Chip. 2018; 18: 3578-87.
3. Fogel JL, Thein TZT, Mariani FV. Use of LysoTracker to detect programmed cell death in embryos and differentiating embryonic stem cells. JoVE (Journal of Visualized Experiments). 2012: e4254.
4. Anderson KE, To M, Olzmann JA, Nomura DK. Chemoproteomics-enabled covalent ligand screening reveals a thioredoxin-caspase 3 interaction disruptor that impairs breast cancer pathogenicity. ACS chemical biology. 2017; 12: 2522-8.
5. Smolkova B, Lunova M, Lynnyk A, Uzhytchak M, Churpita O, Jirsa M, et al. Non-thermal plasma, as a new physicochemical source, to induce redox imbalance and subsequent cell death in liver cancer cell lines. Cell Physiol Biochem. 2019; 52: 119-40.
