# Supplementary material for: Accelerating precision anti-cancer therapy by time-lapse and label-free 3D tumor slice culture platform
Source: Theranostics. 2021 Sep 13;11(19):9415–30. doi: 10.7150/thno.59533 (PMC8490519; doi:10.7150/thno.59533)
Supplement: Supplementary file 7 — Supplementary tables 6-8. [file thnov11p9415s7.pdf]

Supplementary Table 6-8

Table S6. Antibodies used in this research.

Antibodies	Brand	Catalog	Host species	Dilution
Cleaved Caspase-3 (Asp175) Antibody	CST	9661S	Rabbit	1:100
phospho-γH2AX	CST	#2577s	Rabbit	1:100
Anti-Ki67 antibody	Abcam	ab16667	Rabbit	1:200
Desmin	Santa Cruz	sc-23879	Mouse	1:50
anti-Vimentin	Abcam	ab201637	Rat	1:200
Anti-CK7	Abcam,	ab9021	Mouse	1:200
E-cadherin	CST	#3195s	Rabbit	1:100
F4/80 (D2S9R) XP® Rabbit mAb	CST	70076s	Rabbit	1:100
Anti-mouse-CD279 (a-PD-1,29F.1.A12)	BioLegend	135202	Rat	1:100
Anti-mouse-CD274(a-PD-L1,B7-H1)	BioLegend	124302	Rat	1:100
Anti-mouse- CD3e	eBioscience	16-0031-82	Armenian hamster	1:200
Anti-mouse- CD8a	eBioscience	42-0081-82	Rat	1:200
CD45 (D3F8Q) Rabbit mAb	CST	#70257	Rabbit	1:100
Anti CD3	Abcam	ab5690	Rabbit	1:200
CD4 Monoclonal Antibody (GK1.5)	eBioscience	14-0041-82	Rat	1:200
Anti-human PD-L1 antibody	GeneTex	GTX104763	Rabbit	1:100
IgG4, Kappa from human myeloma plasma	Sigma	I4639-1MG	human	
Pembrolizumab (anti-human PD-1)	Selleckchem	A2005-5MG	human	
IgG1, Kappa from human myeloma plasma	Sigma	I5154-1MG	human	
Durvalumab (anti-human PD-L1)	Selleckchem	A2013 -5MG	human	

Table S7. Kits and Chemicals used in this research.

Items	Brand	Catalog
Recombinant Murine interleukin-2	Peprotech	212-12
QuantiTect Reverse Transcription Kit (400)	Qiagen	205314
FastStart Universal SYBR Green Master (Rox)	Roche	4913914001
SPLInsert™ Standing, 12 Inserts/24 well plate, PET	SPL Life Sciences	37524
Cultrex 3-D Culture Matrix™ Rat Collagen I	Sigma	3447-020-01

Table S8. The primers used in this research.

**Primers for qRT-PCR analysis of human immune genes**		
18s Forward: TCGTCTTCGAAACTCCGACT	BGI	N/A
18s Reverse: CGCGGTTCTATTTTGTTGGT	BGI	N/A
Gzmb Forward: CCCTGGGAAAACACTCACACA;	BGI	N/A
Gzmb Reverse: GCACAACTCAATGGTACTGTCG	BGI	N/A
Prf1 Forward: GACTGCCTGACTGTCGAGG;	BGI	N/A
Prf1 Reverse: TCCCGGTAGGTTTGGTGGAA;	BGI	N/A
PDCD1 Forward: CCAAGGCGCAGATCAAAGAGA;	BGI	N/A
PDCD1 Reverse: AGGACCCAGACTAGCAGCA	BGI	N/A
